# Association between antimicrobial regimens and 28-day mortality in patients with *Stenotrophomonas maltophilia* infection: a propensity score-weighted analysis

**DOI:** 10.1128/spectrum.01182-26

**Published:** 2026-07-21

**Authors:** Shumin Gu, Xinyu Liu, Wenjing Lu, Shuzheng Du, Longhua Hu, Yunwei Zheng, Xingwei Cao, Yaping Hang, Zihan Li, Lihua Huang, Qiaoshi Zhong

**Affiliations:** 1 Department of Clinical Laboratory, Jiangxi Province Key Laboratory of Immunology and Inflammation, Jiangxi Provincial Clinical Research Center for Laboratory Medicine, The Second Affiliated Hospital, Jiangxi Medical College, Nanchang University 74653, Nanchang, China; 2 School of Clinical Medicine, Jiangxi University of Chinese Medicine, Nanchang, China; 3 College of Anesthesiology, Henan Medical University 91593, Xinxiang, China; bioMerieux Inc, Salt Lake City, Utah, USA; Barnes-Jewish Hospital, St. Louis, Missouri, USA

**Keywords:** *Stenotrophomonas maltophilia*, antimicrobial therapy, combination therapy, 28-day mortality, cefoperazone-sulbactam, trimethoprim-sulfamethoxazole

## Abstract

*Stenotrophomonas maltophilia (S. maltophilia*) infections carry a high mortality risk, and optimal therapy remains uncertain. We compared commonly used regimens for 28-day outcomes. We conducted a retrospective cohort study of adult inpatients with *S. maltophilia* infection between 2020 and 2023. Definitive regimens included fluoroquinolone (FQ) monotherapy, cefoperazone-sulbactam (CSL) monotherapy, CSL plus FQ, and CSL plus trimethoprim-sulfamethoxazole (TMP-SMX). Confounding was addressed using propensity score-based inverse probability of treatment weighting (IPTW). The primary outcome was 28-day all-cause mortality assessed with weighted Cox models and Kaplan-Meier analyses. Overall, 28-day mortality was 31% (81/261). In the IPTW-weighted analysis, shock and chronic respiratory failure were associated with higher 28-day mortality, whereas combination therapy, compared with monotherapy, was associated with lower mortality. In regimen-specific analyses, FQ monotherapy showed the highest mortality estimate. CSL monotherapy and CSL plus FQ were associated with lower mortality than FQ monotherapy, while CSL plus FQ did not differ significantly from CSL monotherapy. CSL plus TMP-SMX was associated with lower mortality than both monotherapy regimens and had numerically lower mortality than CSL plus FQ (7% vs 19%), although this difference was not statistically significant. In this IPTW-adjusted cohort, combination therapy, compared with monotherapy, was associated with better 28-day survival outcomes. CSL-based regimens were associated with more favorable outcomes than FQ monotherapy, with CSL plus TMP-SMX showing the highest observed survival, although its advantage over CSL plus FQ was not statistically significant. Prospective multicenter studies are warranted to confirm these observations.

**IMPORTANCE:**

*Stenotrophomonas maltophilia* is a difficult-to-treat hospital pathogen that can cause severe infections with high mortality, yet the best antibiotic strategy remains uncertain. In this study, we compared commonly used treatment regimens in hospitalized adults and found that patients receiving combination therapy had better 28-day survival than those receiving monotherapy. Fluoroquinolone monotherapy was associated with the poorest outcomes, whereas cefoperazone-sulbactam-based regimens generally performed better. Among the regimens evaluated, cefoperazone-sulbactam plus trimethoprim-sulfamethoxazole showed the most favorable observed survival, although this advantage over cefoperazone-sulbactam plus fluoroquinolone was not statistically significant. These findings provide real-world evidence to help guide treatment decisions for *S. maltophilia* infections, especially in settings where access to newer antimicrobial agents is limited, and highlight the need for prospective multicenter studies.

## INTRODUCTION

*Stenotrophomonas maltophilia (S. maltophilia*) is an aerobic, gram-negative, opportunistic pathogen (1) and is frequently recovered from healthcare reservoirs such as water outlets, endoscopes, nebulizers, and dialysis fluids (2). It can cause infections in vulnerable populations, particularly immunocompromised individuals, including patients with cystic fibrosis or secondary immunosuppression due to malignancy or other clinical conditions (3). These infections encompass a wide spectrum of clinical syndromes. Respiratory tract and bloodstream infections are most common, while endocarditis, as well as infections of the eye, liver, nervous system, urinary tract, bone, soft tissues, and gastrointestinal tract, have also been described (4). This broad spectrum of clinical manifestations has emerged as a growing concern in healthcare settings. Over recent decades, the incidence of *S. maltophilia* infections has steadily increased (5), and these infections are associated with considerable mortality. Crude mortality rates have reached as high as 69% (6), with immunosuppression, pulmonary infection, septic shock, and intensive care unit (ICU) admission most frequently reported as independent predictors of death (7, 8).

The high mortality associated with these infections is further complicated by significant therapeutic challenges. Therapeutic management of *S. maltophilia* is particularly difficult because of multiple intrinsic and acquired resistance mechanisms. Reduced outer-membrane permeability and active efflux pumps contribute to resistance to several major antibiotic classes, including cephalosporins, aminoglycosides, and tetracyclines (9, 10). Importantly, *S. maltophilia* is also intrinsically resistant to broad-spectrum carbapenems—agents such as imipenem and meropenem that are generally considered last-line therapies for multidrug-resistant gram-negative infections (11). These resistance features substantially limit empirical and definitive treatment options and make regimen selection highly challenging in clinical practice. Given these characteristics, *S. maltophilia* has been designated a priority multidrug-resistant pathogen in healthcare settings where therapeutic options remain limited (4).

In response to these therapeutic challenges, the Infectious Diseases Society of America (IDSA) has developed specific treatment recommendations. Currently, the IDSA recommends treatment strategies that include either dual-agent therapy with active drugs (e.g., cefiderocol, minocycline, trimethoprim-sulfamethoxazole [TMP-SMX], or levofloxacin) or the combination of ceftazidime-avibactam plus aztreonam (12). However, the guidance simultaneously emphasizes that comparative clinical evidence remains limited, and the incremental benefit of combination therapy over monotherapy has not been definitively established (12). Notably, among the recommended therapeutic options, the availability and affordability of newer antimicrobial agents, such as cefiderocol and ceftazidime-avibactam, remain uneven across regions. In resource-limited settings, their clinical use may be constrained by regulatory approval, hospital formulary access, cost, and reimbursement. Therefore, in many real-world clinical settings, minocycline, TMP-SMX, and levofloxacin remain important, commonly used agents for the treatment of *S. maltophilia* infections.

Among fluoroquinolones (FQs), levofloxacin is generally the most commonly considered representative agent for *S. maltophilia* (12). However, ciprofloxacin and moxifloxacin may also be encountered in selected patients in real-world clinical practice, and previous studies have evaluated or discussed these agents as fluoroquinolones in the context of *in vitro* activity or clinical relevance (13, 14). Therefore, analyzing fluoroquinolone-containing regimens as a treatment category in retrospective real-world studies has a practical basis. Cefoperazone-sulbactam (CSL), a β-lactam/β-lactamase inhibitor combination, is widely used in parts of Asia for gram-negative infections, particularly in healthcare settings where access to newer antimicrobial agents is limited (15). Although CSL is not a central agent in current IDSA guidance for *S. maltophilia* infections and its availability and routine use vary across regions, it remains clinically relevant in settings where it is readily available and commonly prescribed for multidrug-resistant gram-negative infections (16). Evidence regarding CSL-based regimens for *S. maltophilia* infections remains limited. Therefore, evaluating CSL monotherapy and CSL-based combination regimens in real-world clinical cohorts may provide useful clinical information for regions where CSL is part of routine antimicrobial practice.

For decades, research and infection-control efforts have primarily focused on the traditional ESKAPE pathogens (17), whereas *S. maltophilia*, despite being similarly challenging in terms of antimicrobial resistance and therapeutic decision-making, remains relatively understudied. This disparity highlights an imbalance in research attention and underscores the need for dedicated investigations into effective therapeutic strategies for this pathogen. To contribute to this understudied area and provide evidence-based information for clinicians, this study aimed to compare the association of commonly used therapeutic regimens—including FQ monotherapy, CSL monotherapy, CSL plus FQ, and CSL plus TMP-SMX—with 28-day outcomes in patients with *S. maltophilia* infection.

## MATERIALS AND METHODS

### Study design and patient population

**Fig 1:**
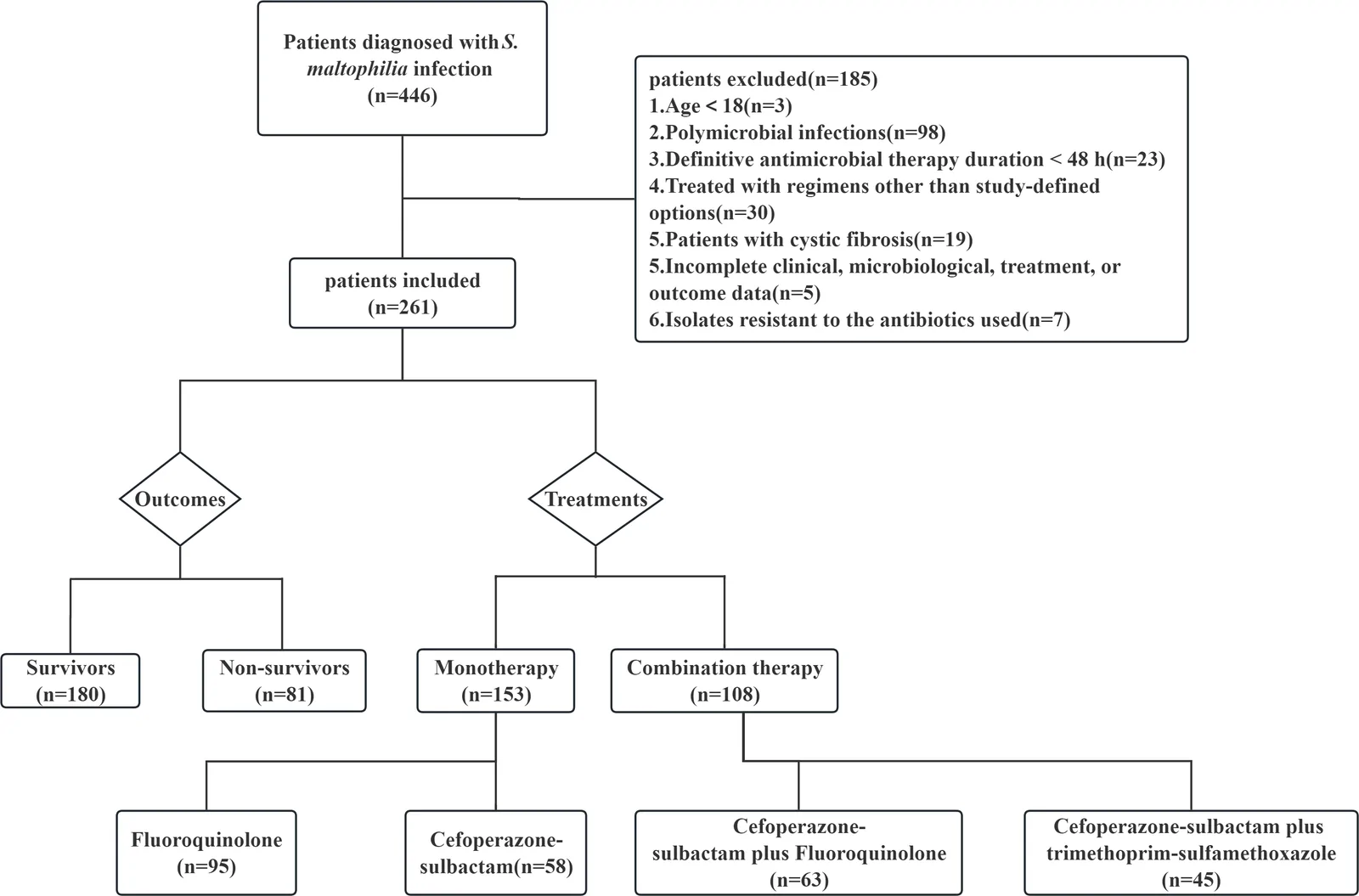
Flow chart of patients selected.

This retrospective study was conducted at a 3,600-bed tertiary general hospital in southern China. Adult inpatients diagnosed with *S. maltophilia* infection between 1 January 2020 and 31 December 2023 were screened. Only the first episode of *S. maltophilia* infection during the study period was considered for analysis. Patients were excluded if they were younger than 18 years, had polymicrobial infections, received definitive antimicrobial therapy for less than 48 h, received regimens other than the four study-defined regimens, had cystic fibrosis, had incomplete clinical, microbiological, treatment, or outcome data, or had isolates resistant *in vitro* to the antibiotics used for definitive therapy. Patients with structural lung diseases other than cystic fibrosis were not excluded. The detailed patient selection process is shown in Fig. 1.

### Data collection

Clinical data were extracted from the Laboratory Information System and Hospital Information System. The data set included demographic characteristics, microbiological details, infection types, length of hospital stay, time of infection onset, as well as clinical information such as ICU stay, parenteral nutrition, surgical procedures, hemodialysis, and invasive interventions related to the site of infection. Comorbidities recorded were diabetes, hypertension, cerebrovascular disease, chronic liver disease, cardiovascular disease, solid tumors, hematologic malignancies, chronic heart failure, chronic respiratory failure, structural lung disease, chronic renal failure, major bleeding, and immunocompromised status. Clinical conditions at infection onset (arrhythmia, shock, hypoalbuminemia, and coma), definitive antimicrobial treatment regimens, and time-related variables potentially affecting treatment timing were also collected. These time-related variables included time from hospital admission to first positive culture collection, time from culture collection to antimicrobial susceptibility reporting, days of use of any definitive-regimen agent before susceptibility reporting, and whether any definitive-regimen agent was initiated before susceptibility reporting. In addition, Sequential Organ Failure Assessment (SOFA) scores at infection onset and survival status at 28 days after infection onset were recorded.

### Definitions

Combination therapy was defined as the use of two agents with documented *in vitro* activity against the target pathogen, initiated concurrently or within 48 h of each other and overlapping for at least 48 h; patients in whom the second active agent was added more than 48 h after initiation of the first active agent were not classified as receiving combination therapy. Monotherapy was defined as the use of a single active agent. Because the exact clinical onset of infection could not be reliably determined retrospectively, the onset of *S. maltophilia* infection was operationally defined as the date of collection of the first positive culture. Sex was recorded as male or female in the electronic medical record. Chronic respiratory failure was defined as a documented diagnosis of chronic hypoxemic or hypercapnic respiratory failure before the onset of *S. maltophilia* infection. Baseline respiratory support, including oxygen therapy via nasal cannula or face mask, tracheostomy, or endotracheal intubation with invasive mechanical ventilation, was reviewed in the medical record. Acute oxygen supplementation or mechanical ventilation initiated only for the index infection was not classified as chronic respiratory failure. Structural lung disease was defined as chronic pulmonary disease with documented structural abnormalities or chronic airway/lung remodeling in the medical record, including chronic obstructive pulmonary disease, bronchiectasis, and interstitial lung disease. Site-related invasive procedures were operationally defined according to the infection site and referred to relevant devices/procedures that had been established prior to infection onset and remained in place at the time of onset: endotracheal intubation or tracheostomy for pulmonary infections; indwelling urinary catheterization for urinary tract infections; abdominal drainage catheter placement for intra-abdominal infections; and biliary drainage catheter placement for biliary tract infections. Major bleeding was defined according to the International Society on Thrombosis and Haemostasis (ISTH) criteria as fatal bleeding and/or symptomatic bleeding in a critical area or organ, including intracranial hemorrhage, and/or bleeding associated with a hemoglobin decrease of ≥2 g/dL or transfusion of ≥2 units of red blood cells/whole blood. Patients were considered immunosuppressed if they had active immunosuppression during the index hospitalization and before or at the onset of *S. maltophilia* infection, including active cancer chemotherapy, systemic corticosteroid therapy equivalent to ≥20 mg prednisone per day for ≥14 days or a cumulative dose of ≥600 mg, biological immunomodulators, or other systemic immunosuppressive medications. All-cause mortality was defined as death from any cause within 28 days of the onset of *S. maltophilia* infection.

### Diagnostic criteria for *S. maltophilia* infection

In this study, pulmonary infections caused by *S. maltophilia* were diagnosed as pneumonia based on a combination of clinical, radiographic, oxygenation, and microbiological criteria. At our institution, quantitative culture was performed for bronchoalveolar lavage (BAL) specimens, whereas other respiratory specimens were generally reported semi-quantitatively. Therefore, respiratory culture results were interpreted according to specimen type and were not used as a stand-alone diagnostic criterion. For BAL specimens, quantitative results were considered as supportive microbiological evidence, with a threshold of 10⁴ CFU/mL (18). For non-BAL respiratory specimens, including sputum and endotracheal aspirates, semi-quantitative organism burden was considered as supportive evidence and interpreted in conjunction with specimen quality and the overall clinical context. For sputum specimens, only qualified samples were considered, defined as >25 neutrophils/LPF and <10 epithelial cells/LPF, or a neutrophil-to-epithelial cell ratio >2.5:1 (19). Pneumonia was further defined by the presence of a new or progressive pulmonary infiltrate on imaging, clinical findings such as fever (≥38°C) or hypothermia (≤36.5°C), leukocytosis (≥12,000/µL) or leukopenia (≤4,000/µL), purulent respiratory secretions, a positive respiratory specimen for *S. maltophilia*, and evidence of impaired oxygenation (20). Urinary tract infection was defined as isolation of *S. maltophilia* from midstream urine at ≥10⁵ CFU/mL, in conjunction with compatible urinary symptoms, pyuria, fever, or systemic signs of infection without a more likely alternative source (21), and clinician-documented diagnosis or treatment of urinary tract infection. Positive urine culture alone was not considered sufficient for the diagnosis of urinary tract infection, and asymptomatic bacteriuria was not classified as *S. maltophilia* infection. Other types of infection were defined according to the CDC/NHSN criteria (22).

### Antimicrobial treatment

Treatment referred to definitive therapy, defined as the administration of agents to which *S. maltophilia* was susceptible. Four regimens were analyzed: FQ monotherapy (levofloxacin, moxifloxacin, or ciprofloxacin), CSL monotherapy, CSL plus an FQ, and CSL plus TMP-SMX. Definitive therapy was defined as the administration of agents to which the *S. maltophilia* isolate was susceptible *in vitro* and was initiated within 2 days before or up to 2 days after the date on which the first culture was reported positive for *S. maltophilia*. Antimicrobial therapy initiated before culture positivity was considered empiric at the time of administration but was retrospectively classified as definitive therapy if the agent was subsequently confirmed to have *in vitro* activity against the patient’s *S. maltophilia* isolate and met the prespecified timing and duration criteria. The treatment course was defined as the duration of antimicrobial therapy directed against *S. maltophilia*, from initiation of the first agent subsequently confirmed to have *in vitro* activity against the isolate to discontinuation of anti-*S*. *maltophilia* therapy, death, or hospital discharge, whichever occurred first. Patients were required to receive definitive antimicrobial therapy for at least 48 h and for at least 70% of the treatment course directed against *S. maltophilia*. The dosing regimens reflected physician-prescribed treatment in routine clinical practice and were adjusted according to local drug labeling, renal function, infection severity, infection site, and individual patient tolerance. Except for TMP-SMX, which was administered orally, the study antibiotics were administered intravenously. The dosing regimens during treatment were as follows: levofloxacin 500 mg every 12 h, ciprofloxacin 400 mg every 8 h, moxifloxacin 400 mg every 24 h, and CSL most commonly at 9 g per 24 h in three divided doses, with less frequent use of 6 or 12 g per 24 h. TMP-SMX was administered orally as 0.96 g per dose every 8 or 12 h, corresponding to trimethoprim 160 mg plus sulfamethoxazole 800 mg per dose, with dose adjustment based on renal function when clinically indicated. Antimicrobial susceptibility breakpoints were interpreted according to the Clinical and Laboratory Standards Institute (CLSI) guidelines. Since CLSI does not provide breakpoints for *S. maltophilia* with moxifloxacin, ciprofloxacin, or CSL, ciprofloxacin was interpreted using the criteria for non-Enterobacteriaceae (23), moxifloxacin was interpreted according to ciprofloxacin breakpoints, and CSL was interpreted based on cefoperazone breakpoints for non-Enterobacteriaceae (24).

### Statistical analysis

Categorical variables were summarized as frequencies and percentages and compared using the *χ*^2^ test. To reduce measured baseline confounding, propensity scores were estimated using a multinomial logistic regression model with treatment group as the dependent variable. Covariates for the propensity score model were selected based on clinically relevant baseline variables with observed imbalance across treatment groups, as shown in Table S1. The propensity score model included respiratory system SOFA score ≥2, cardiovascular system SOFA score ≥2, immunocompromised condition, and coma. Inverse probability of treatment weighting (IPTW) was then applied to construct a weighted survival data set, with weights defined as the inverse of the estimated probability of receiving the treatment actually administered. The distribution of IPTW weights was examined, and to mitigate the influence of extreme weights, weights were truncated (winsorized) at the 1st and 99th percentiles (Tables S2 and S3; Fig. S1 and S2). Covariate balance after weighting was assessed using standardized mean differences (SMDs), with SMD < 0.1 indicating adequate balance (Fig. S3 and S4). In the weighted data set, associations between treatment regimens and 28-day all-cause mortality were evaluated using a multivariable Cox proportional hazards regression model. IPTW-adjusted pairwise post-hoc comparisons between treatment groups were performed based on the weighted Cox model, with *P* values corrected using the Tukey method. Survival distributions were estimated using weighted Kaplan-Meier curves. Sensitivity analyses were performed to assess the robustness of the findings: for the two-group comparison (combination therapy vs monotherapy), stabilized IPTW weights were used to refit the weighted Cox model (Table S4); for the four-regimen analysis, the weighted Cox model was refitted using untruncated (non-winsorized) IPTW weights (Table S5). To assess the robustness of findings to potential treatment-allocation bias related to antimicrobial resistance, we performed an additional resistance-restricted sensitivity analysis. Because the evaluated regimens consisted of FQ monotherapy, CSL monotherapy, CSL plus FQ, and CSL plus TMP-SMX, eligibility for this analysis was defined as documented *in vitro* susceptibility to all three study drugs/classes, namely CSL, FQ, and TMP-SMX, and absence of documented contraindications to these agents, such as drug allergy (Table S6). Within this restricted cohort, propensity scores were re-estimated, IPTW weights were recalculated, covariate balance was reassessed using standardized mean differences (Fig. S5), and weighted Cox proportional hazards models were refitted for both the comparison of combination therapy vs monotherapy and the four-regimen comparison (Table S7). Given the retrospective design, a post hoc power calculation was performed for the primary comparison between monotherapy and combination therapy based on short-term mortality estimates reported in prior studies of *S. maltophilia* infection (25, 26). Assuming mortality rates of 40% for monotherapy and 25% for combination therapy, the available sample size provided an estimated power of approximately 73% at a two-sided α level of 0.05. All analyses were conducted in R (version 4.3.3), and a two-sided *P* < 0.05 was considered statistically significant.

## RESULTS

### Distribution of infection types in *S. maltophilia*

**Fig 2:**
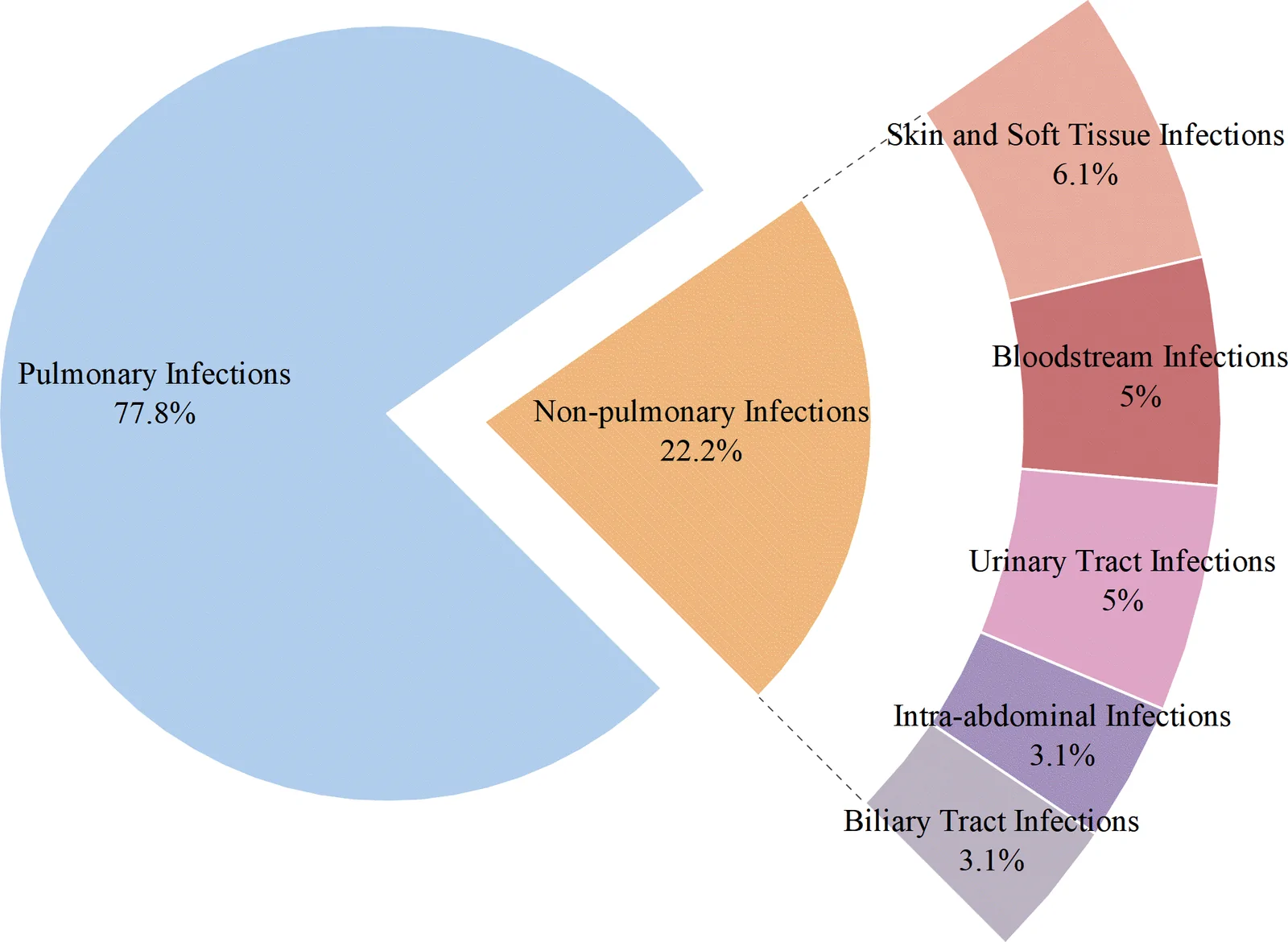
Distribution of pulmonary and non-pulmonary infection types in patients with *S. maltophilia*. Each patient was classified according to a single primary infection-site category for analysis.

A total of 261 patients with *S. maltophilia* infection were included in the analysis. Pulmonary infections were the most common, accounting for 77.8% of cases. Non-pulmonary infections comprised 22.2%, with skin and soft tissue infections being the most frequent (6.1%), followed by bloodstream and urinary tract infections (both 5.0%; Fig. 2).

### Demographic and clinical characteristics of the study population

Among the 261 included patients, the 28-day all-cause mortality rate was 31% (81/261). Combination therapy was administered to 41% of patients (108/261), while 59% received monotherapy (153/261). Compared with non-survivors, survivors were more likely to be aged ≥60 years (*P* < 0.001) and to have received combination therapy (*P* < 0.001). In contrast, non-survivors more frequently required ICU admission (*P* = 0.001) and underwent invasive procedures at the infection site (*P* = 0.049). Additionally, chronic respiratory failure (*P* < 0.001), major bleeding (*P* = 0.047), and pulmonary infection (*P* = 0.010) were more common among non-survivors, along with a higher prevalence of shock (*P* < 0.001) and coma (*P* = 0.029) at the onset of infection (Table 1).

**TABLE 1:** Characteristics of patients in the non-survival and survival groups^*c*^^,^^*d*^^,*e*^

Variables *N* (%)	Overall *N* = 261	Survival *N* = 180	Non-survival *N* = 81	*P* value
**Older age (≥60 years)**	153 (59)	123 (68)	30 (37)	<0.001
**Sex, male**	188 (72)	130 (72)	58 (72)	0.918
**Hospitalization for >30 days prior to infection**	105 (40)	75 (42)	30 (37)	0.480
**ICU admission**	107 (41)	62 (34)	45 (56)	0.001
**Parenteral nutrition**	69 (26)	42 (23)	27 (33)	0.090
**Surgical operation^*a*^**	63 (24)	39 (22)	24 (30)	0.164
**Hemodialysis^*a*^**	28 (11)	19 (11)	9 (11)	0.893
**Invasive procedures related to the site of infection**	144 (55)	92 (51)	52 (64)	0.049
**Pulmonary infection**	203 (78)	132 (73)	71 (88)	0.010
**Co-morbidities**				
Diabetes mellitus	52 (20)	33 (18)	19 (23)	0.338
Hypertension	103 (39)	74 (41)	29 (36)	0.417
Cerebrovascular disease	57 (22)	37 (21)	20 (25)	0.454
Chronic hepatic disease	78 (30)	53 (29)	25 (31)	0.817
Cardiovascular disease	81 (31)	57 (32)	24 (30)	0.742
Solid tumor	56 (21)	36 (20)	20 (25)	0.393
Hematologic malignancy	15 (6)	11 (6)	4 (5)	0.929^*b*^
Chronic heart failure	65 (25)	42 (23)	23 (28)	0.382
Chronic respiratory failure	125 (48)	70 (39)	55 (68)	<0.001
Structural lung disease	109 (42)	69 (38)	40 (49)	0.094
Chronic renal failure	79 (30)	50 (28)	29 (36)	0.192
Major bleeding	49 (19)	28 (16)	21 (26)	0.047
Immunocompromised condition	82 (31)	56 (31)	26 (32)	0.874
**Diagnosis at the onset of *S. maltophilia* infection**				
Arrhythmia	55 (21)	36 (20)	19 (23)	0.526
Shock	49 (19)	20 (11)	29 (36)	<0.001
Hypoalbuminemia	211 (81)	141 (78)	70 (86)	0.125
Coma	53 (20)	30 (17)	23 (28)	0.029
**SOFA scoring**				
Respiratory system score ≥ 2	140 (54)	86 (48)	54 (67)	0.005
Cardiovascular system score ≥ 2	38 (15)	25 (14)	13 (16)	0.647
Neurological systems score ≥ 2	88 (34)	49 (27)	39 (48)	0.001
Liver system score ≥ 2	35 (13)	22 (12)	13 (16)	0.401
Kidney system score ≥ 2	54 (21)	35 (19)	19 (23)	0.459
Coagulation system score ≥ 2	59 (23)	36 (20)	23 (28)	0.134
**Treatment approach**				<0.001
Combination therapy	108 (41)	93 (52)	15 (19)	–^*f*^
Monotherapy	153 (59)	87 (48)	66 (81)	–

^*a*^ Within the last 30 days.

^*b*^ *χ*^2^ test with continuity correction.

^*c*^ SOFA, Sequential Organ Failure Assessment.

^*d*^ Structural lung disease was defined as chronic obstructive pulmonary disease, bronchiectasis, or interstitial lung disease documented in the medical record.

^*e*^ Bold terms indicate category headings.

^*f*^ “–”, not applicable.

### IPTW-adjusted association between treatment approach and 28-day mortality

**Fig 3:**
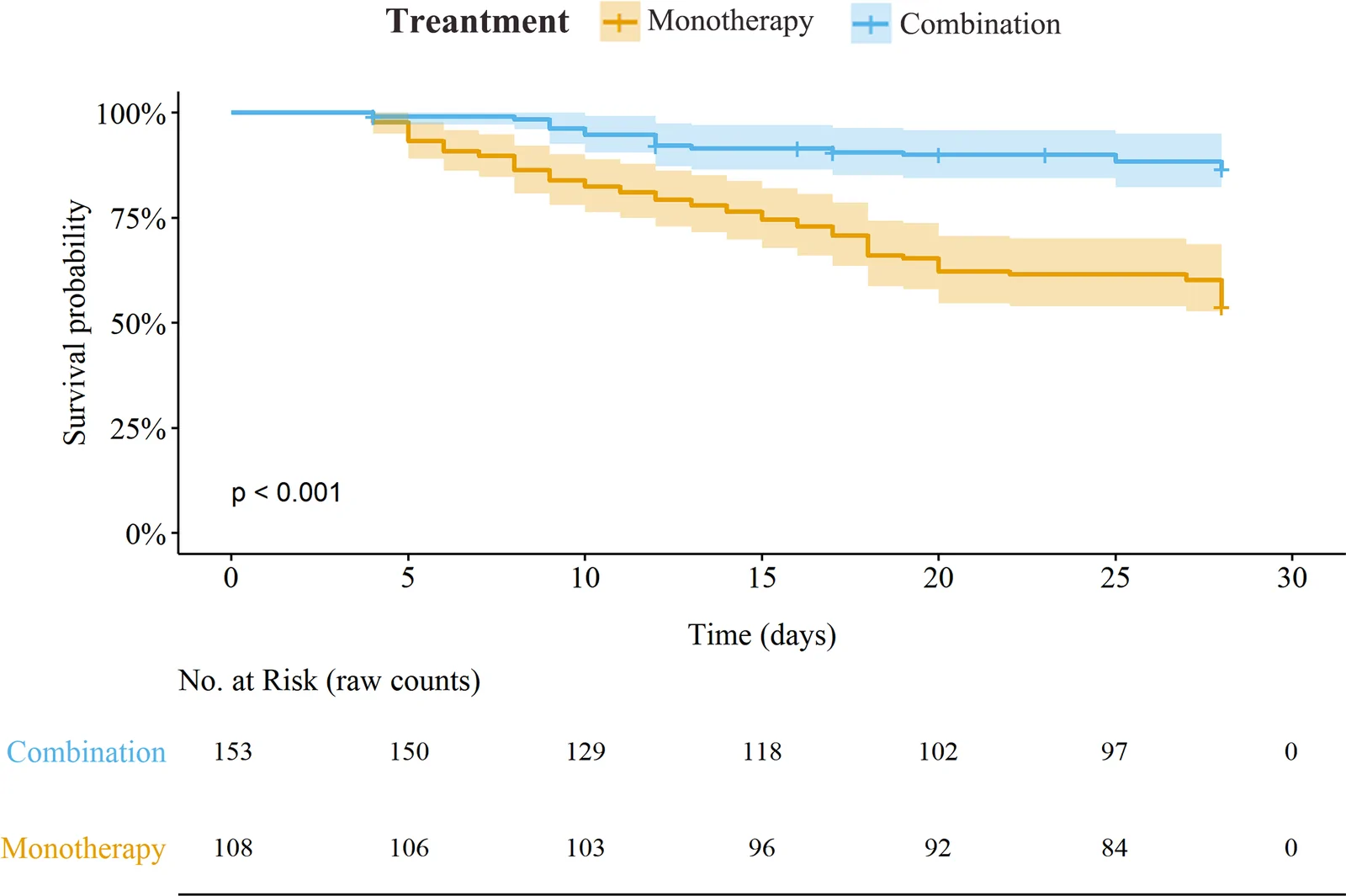
IPTW-adjusted Kaplan-Meier 28-day survival: combination vs monotherapy in *S. maltophilia.*

Propensity score-based IPTW was applied to improve balance in measured baseline characteristics between the monotherapy and combination therapy groups. After weighting, all covariates were well balanced (all SMDs < 0.1; Fig. S3). In the IPTW-weighted Cox model (Table 2), shock (HR = 2.484, 95% CI: 1.390–4.438, *P* = 0.002) and chronic respiratory failure (HR = 2.402, 95% CI: 1.352–4.269, *P* = 0.002) were associated with higher 28-day all-cause mortality in patients with *S. maltophilia* infection. In contrast, combination therapy (vs monotherapy) was associated with a lower hazard of 28-day all-cause mortality (HR = 0.235, 95% CI: 0.128–0.434, *P* < 0.001). This association remained consistent in the sensitivity analysis using stabilized IPTW weights (Table S4) and in the additional resistance-restricted sensitivity analysis (Table S7). Among the 208 patients included in the restricted cohort, combination therapy remained associated with lower 28-day all-cause mortality compared with monotherapy (HR = 0.236, 95% CI: 0.118–0.472, *P* < 0.001). Consistent with these findings, IPTW-adjusted Kaplan-Meier curves showed higher 28-day survival probabilities in the combination therapy group than in the monotherapy group (weighted log-rank *P* < 0.001; Fig. 3).

**TABLE 2:** IPTW-weighted Cox model evaluating the association between treatment approach and 28-day mortality in patients with *S. maltophilia* infection^*a*^

Variable	Multivariable analysis HR (95% CI)	*P* value
Older age ( 60 years)	0.910 (0.550–1.504)	0.714
SOFA neurological score ≥ 2	1.047 (0.596–1.838)	0.870
Invasive procedures related to the site of infection	1.074 (0.627–1.841)	0.792
Shock	2.484 (1.390–4.438)	0.002
Chronic respiratory failure	2.402 (1.352–4.269)	0.002
Treatment approach (combination therapy vs monotherapy)	0.235 (0.128–0.434)	<0.001

^*a*^ HR, hazard ratio; CI, confidence interval; IPTW, inverse probability of treatment weighting; SOFA, Sequential Organ Failure Assessment.

### IPTW-adjusted association between specific antibiotic regimens and 28-day mortality

Building on the comparison between monotherapy and combination therapy, patients were further stratified into four antibiotic regimen groups (Table S6). Time-related variables potentially affecting treatment timing were generally comparable across the four treatment groups (Table S8). IPTW was then re-applied for the four-regimen analysis. Covariate balance was substantially improved after weighting. Most post-weighting SMDs were <0.1, although cardiovascular system SOFA score ≥2 remained marginally above the prespecified threshold (Fig. S4). A weighted Cox proportional hazards model was then constructed to evaluate the association between specific antibiotic regimens and 28-day all-cause mortality (Table 3). Compared with the reference group (CSL monotherapy), FQ monotherapy was associated with a higher hazard of 28-day all-cause mortality (HR = 2.345, 95% CI: 1.325–4.149, *P* = 0.003). In contrast, CSL combined with TMP-SMX was associated with a lower hazard of mortality (HR = 0.158, 95% CI: 0.038–0.653, *P* = 0.010). The combination of CSL and FQ did not show a statistically significant difference compared to CSL monotherapy (HR = 0.572, 95% CI: 0.261–1.252, *P* = 0.162). Similar patterns were observed in sensitivity analyses using untruncated IPTW weights (Table S5) and in the resistance-restricted cohort (Table S7). In the restricted cohort, the direction of regimen-specific estimates remained similar to the primary analysis, with FQ monotherapy showing the highest hazard estimate and CSL plus TMP-SMX showing the lowest hazard estimate.

**TABLE 3:** IPTW-weighted Cox model evaluating the association between specific antibiotic regimens and 28-day mortality^*a*^

Variable	HR (95% CI)	*P* value
Age ≥ 60 years	0.880 (0.521–1.486)	0.633
SOFA (nervous system) ≥2	0.907 (0.514–1.603)	0.738
Intubation	1.125 (0.633–1.998)	0.687
Shock	3.192 (1.730–5.889)	<0.001
Respiratory failure	2.801 (1.586–4.949)	<0.001
Treatment group (ref: CSL)		
FQ	2.345 (1.325–4.149)	0.003
CSL plus FQ	0.572 (0.261–1.252)	0.162
CSL plus TMP-SMX	0.15 8 (0.038–0.653)	0.010

^*a*^ HR, hazard ratio; CI, confidence interval; SOFA, Sequential Organ Failure Assessment; CSL, cefoperazone-sulbactam; FQ, fluoroquinolone; TMP-SMX, trimethoprim-sulfamethoxazole.

### Pairwise comparisons of antibiotic regimens

**Fig 4:**
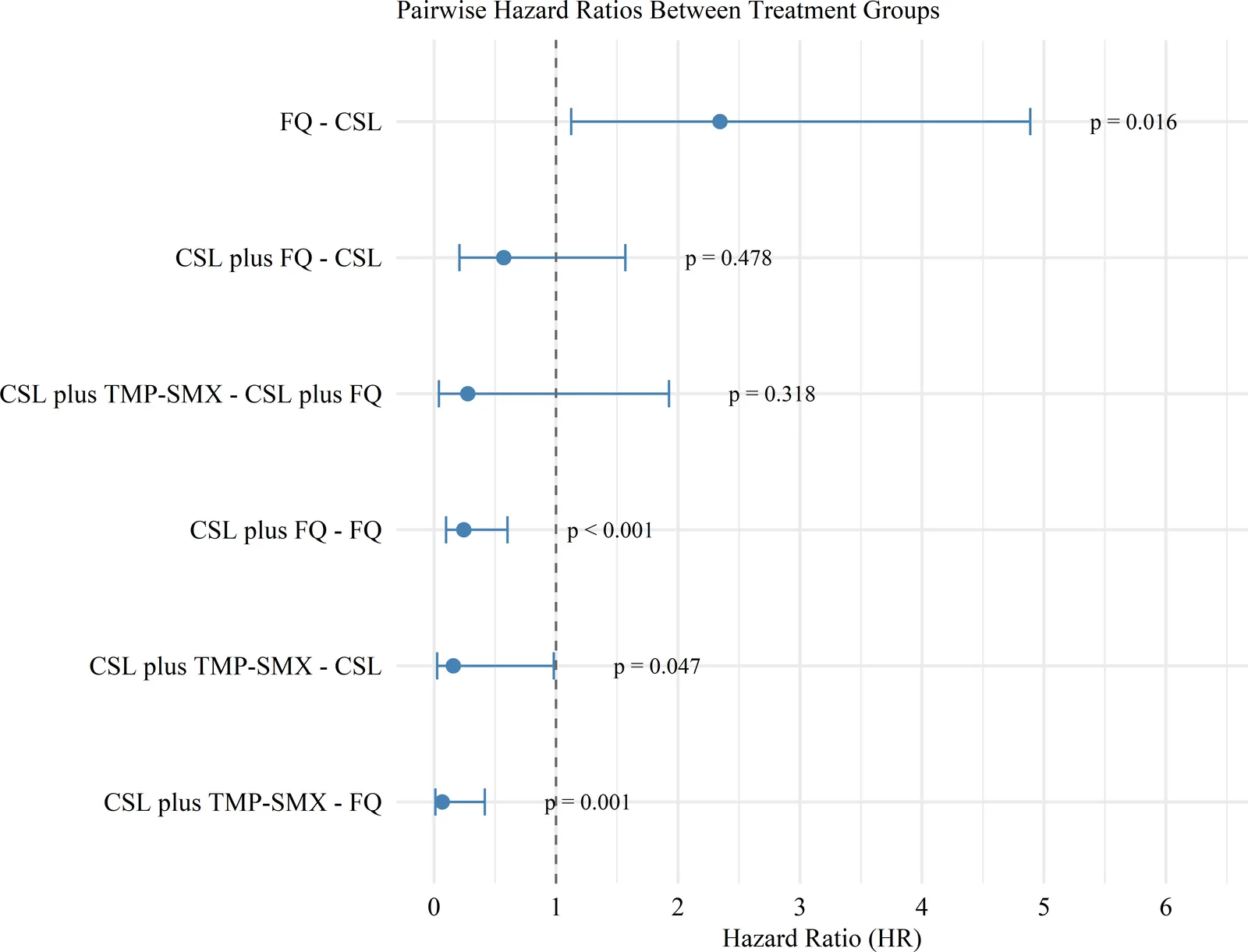
Forest plot of IPTW-adjusted pairwise hazard ratios for 28-day mortality between antibiotic regimens.

To enable direct head-to-head comparisons, IPTW-adjusted pairwise post hoc analyses were performed. CSL monotherapy was associated with a lower hazard of 28-day all-cause mortality than FQ monotherapy (HR = 0.431, 95% CI: 0.202–0.890, *P* = 0.016). CSL plus TMP-SMX showed lower hazard estimates than both monotherapy regimens (CSL plus TMP-SMX vs FQ: HR = 0.072, 95% CI: 0.011–0.423, *P* < 0.001; CSL plus TMP-SMX vs CSL: HR = 0.164, 95% CI: 0.030–0.981, *P* = 0.047). By comparison, CSL plus FQ showed a lower hazard only when compared with FQ monotherapy (HR = 0.241, 95% CI: 0.101–0.604, *P* < 0.001). No significant difference was observed between CSL plus TMP-SMX and CSL plus FQ (HR = 0.280, 95% CI: 0.041–1.930, *P* = 0.318; Fig. 4).

### Kaplan-Meier survival analysis by antibiotic regimens

**Fig 5:**
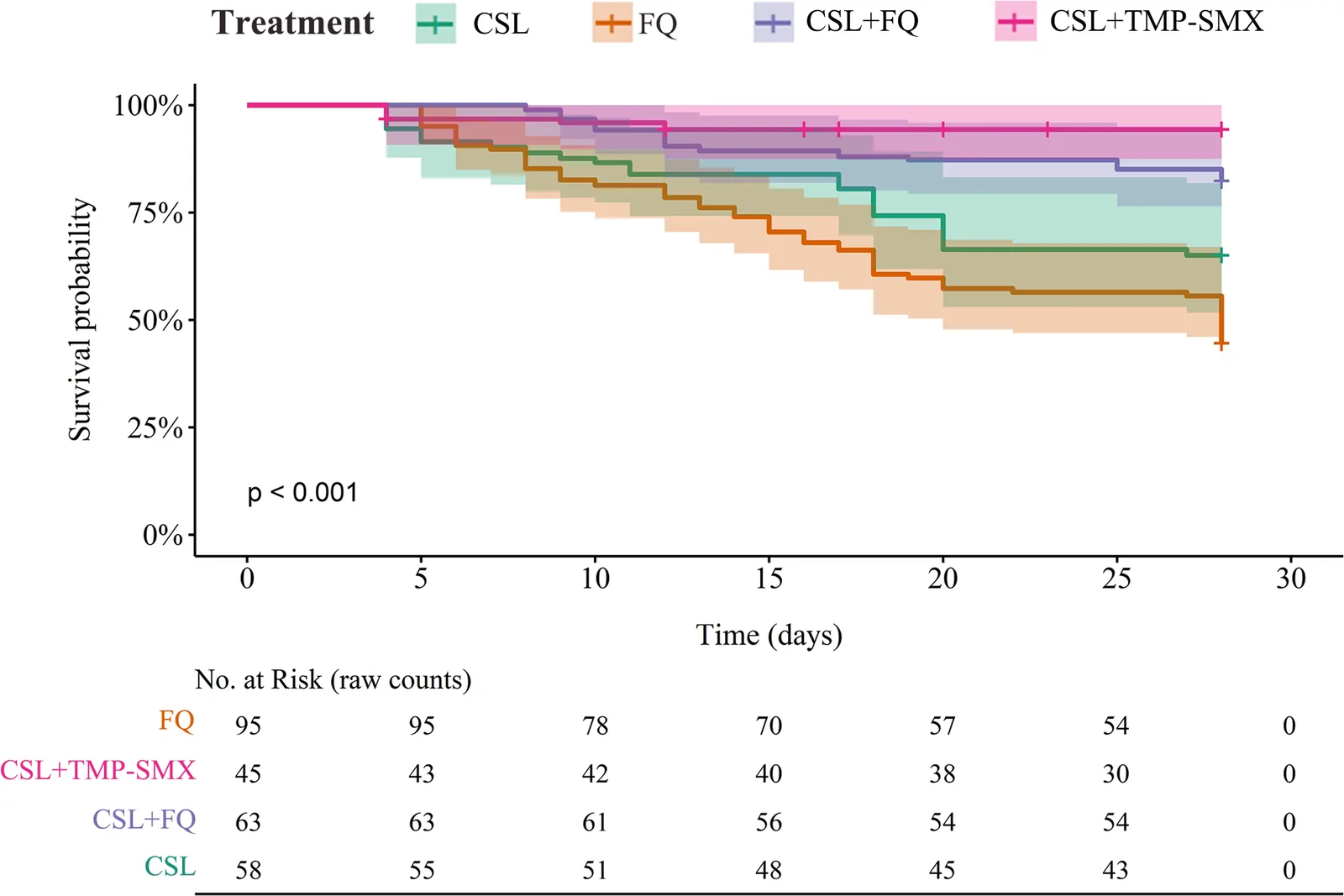
IPTW-adjusted Kaplan-Meier 28-day survival by antibiotic regimen in *S. maltophilia*.

IPTW-adjusted Kaplan-Meier curves were generated to illustrate survival differences among the four treatment groups. As shown in Fig. 5, the 28-day all-cause survival probability differed significantly across regimens (*P* < 0.001, log-rank test). The highest observed survival probability was in the CSL plus TMP-SMX group, whereas the lowest observed survival probability was in the FQ monotherapy group.

## DISCUSSION

*S. maltophilia* is an opportunistic pathogen that can cause infections at multiple sites (4). Although we included a broader spectrum of infection types, pulmonary involvement remained predominant (77.8%), consistent with prior reports in hospitalized patients (3, 27), likely reflecting frequent airway instrumentation and the high prevalence of underlying lung disease in hospitalized patients.

The overall 28-day all-cause mortality in our cohort was 31%. In IPTW-weighted multivariable analysis, shock and chronic respiratory failure were associated with higher 28-day mortality, suggesting that acute physiologic derangement and limited pulmonary reserve may be important markers of poor prognosis. While older age is often associated with worse prognosis (28), the age distribution in our cohort appeared influenced by differences in illness severity and ICU admission, which may have attenuated the expected effect of chronological age.

A key finding was that the treatment approach was independently associated with survival. Combination therapy was associated with significantly lower 28-day mortality than monotherapy (HR = 0.235, 95% CI 0.128–0.434; *P* < 0.001). This is consistent with reports suggesting potential benefit of combination regimens in severe infections and high-risk hosts (25, 29). This observed association may be biologically plausible, given potential synergistic bactericidal activity, mitigation of heteroresistance (30), and complementary tissue penetration that may enhance antimicrobial delivery across heterogeneous microenvironments (31). Despite these mechanistic considerations, clinical outcomes across studies have been inconsistent. Prior studies in critically ill patients with hospital-acquired *S. maltophilia* pneumonia and in immunocompromised hosts reported no mortality benefit vs monotherapy (32, 33). These discrepant findings may reflect residual confounding, including greater illness severity, immunosuppression, and polymicrobial infection in combination-therapy groups. To reduce measured baseline imbalance and attribution bias, we applied IPTW and excluded polymicrobial *S. maltophilia* infections, thereby improving the robustness of the observed treatment-outcome associations.

In regimen-specific comparisons, FQ monotherapy was associated with the highest mortality, whereas CSL monotherapy showed more favorable outcomes. Although CSL is rarely used in Europe and the United States, it remains a pragmatic option in resource-limited Asian settings for multidrug-resistant gram-negative infections (15). Evidence for CSL monotherapy specifically against *S. maltophilia* has been limited; thus, our findings provide clinically relevant real-world data supporting its potential role. CSL may provide more reliable activity in clinical settings where β-lactamase-mediated resistance (6, 28) and biofilm-associated infection are relevant (4), and its time-dependent pharmacodynamics may be advantageous in severe disease when optimized dosing strategies are applied (34, 35). In contrast, FQs require adequate exposure (e.g., AUC/MIC) to achieve efficacy (36). Although all FQ-treated patients in this study had isolates with documented *in vitro* susceptibility to the administered agent, susceptibility alone does not ensure pharmacodynamic target attainment in individual patients. In critically ill patients, exposure variability and safety-limited dose escalation may predispose to suboptimal target attainment (37) and resistance selection (5). These considerations are consistent with the caution against levofloxacin monotherapy in severe *S. maltophilia* infections noted by CLSI (38). However, because MIC distributions, drug exposure data, and AUC/MIC target-attainment analyses were not available, inadequate pharmacodynamic exposure can only be considered a possible contributing factor, rather than a confirmed explanation, for the poorer outcomes observed with FQ monotherapy.

We further assessed CSL-based combinations. CSL plus FQ was associated with lower mortality than FQ monotherapy but was not significantly different from CSL monotherapy, suggesting that the observed association for CSL plus FQ may largely reflect the CSL component. These findings raised the possibility that outcomes may differ according to the partner agent used in CSL-based combinations; therefore, we further evaluated CSL in combination with TMP-SMX. Notably, CSL plus TMP-SMX was associated with the most favorable observed survival; however, its advantage over CSL plus FQ was not statistically significant (mortality 7% vs 19%). TMP-SMX retains high *in vitro* activity against *S. maltophilia* (39, 40) and remains a cornerstone agent in guideline recommendations (41), and contemporary guidance increasingly favors combination strategies for severe infections (12). Consistent with our observations, a cohort study of adults with *S. maltophilia* bacteremia reported improved survival with TMP-SMX plus CSL (28). The lack of a statistically significant difference between CSL-based combinations may reflect limited power and potential effect modification by infection site (42); future prospective studies with site-stratified analyses and optimized dosing assessments are needed to define the optimal regimen in specific clinical contexts.

Several limitations warrant consideration. First, this was a retrospective, single-center study, and residual confounding may persist despite IPTW adjustment. Second, antimicrobial resistance profiles may have influenced treatment allocation. Although a resistance-restricted sensitivity analysis was performed, residual confounding from prior antimicrobial exposure, healthcare exposure, prescribing preference, or undocumented contraindications cannot be excluded. In addition, the restricted cohort had fewer patients and events, particularly in the CSL plus TMP-SMX group, which may have limited the power of regimen-specific comparisons. Third, polymicrobial infections were excluded to reduce attribution bias in evaluating outcomes associated with *S. maltophilia*-directed treatment; however, because *S. maltophilia* is frequently recovered in polymicrobial infection settings, our findings may not be generalizable to polymicrobial infections. Fourth, FQs were analyzed as a single treatment category. Because levofloxacin, ciprofloxacin, and moxifloxacin may differ in antimicrobial activity, pharmacokinetic/pharmacodynamic properties, infection-site applicability, and supporting clinical evidence, this grouping may have introduced heterogeneity; however, the sample size was insufficient for robust analyses of individual FQs. Finally, the non-evaluation of several guideline-recommended regimens due to local drug availability and institutional practice, together with the region-specific use of CSL, may limit the generalizability of our findings. Nevertheless, our findings reflect real-world care in a resource-limited setting and may inform treatment decisions in regions where access to newer antimicrobial agents is restricted.

### Conclusion

In this IPTW-adjusted retrospective cohort of hospitalized patients with *S. maltophilia* infection, 28-day all-cause mortality remained substantial, particularly among patients with shock and chronic respiratory failure. Importantly, combination therapy, compared with monotherapy, was associated with better 28-day survival outcomes. Among the regimens evaluated, CSL-based regimens were associated with more favorable outcomes than FQ monotherapy, with CSL plus TMP-SMX showing the most favorable observed survival, although its advantage over CSL plus FQ was not statistically significant (survival 93% vs 81%). These findings support the consideration of CSL-based regimens as practical treatment options for *S. maltophilia* infection, particularly in resource-limited settings where access to newer antimicrobial agents is restricted. Given the retrospective, single-center design, prospective multicenter studies are warranted to confirm these observations and further evaluate regimen-specific outcomes.

## Supplementary Material

Reviewer comments

## Data Availability

The data sets analyzed during the current study are not publicly available due to patient privacy and institutional restrictions, but are available from the corresponding author on reasonable request and with permission from the Medical Research Ethics Committee of the Second Affiliated Hospital of Nanchang University.

## SUPPLEMENTAL MATERIAL

File S1 spectrum.01182-26-s0001.pdf STROBE checklist.

Fig. S1 spectrum.01182-26-s0002.tif Distribution of IPTW weights (two-group analysis).

Fig. S2 spectrum.01182-26-s0003.tif Distribution of IPTW weights (four-group analysis).

Fig. S3 spectrum.01182-26-s0004.tif Standardized mean differences of baseline covariates between combination therapy and monotherapy groups after IPTW adjustment.

Fig. S4 spectrum.01182-26-s0005.tif Standardized mean differences of baseline covariates between antibiotic regimens after IPTW adjustment.

Fig. S5 spectrum.01182-26-s0006.tif Standardized mean differences after IPTW adjustment in the resistance-restricted sensitivity analysis cohort.

Supplemental tables spectrum.01182-26-s0007.pdf Tables S1 to S8.

OPEN PEER REVIEW reviewer-comments.pdf An accounting of the reviewer comments and feedback.

ASM does not own the copyrights to Supplemental Material that may be linked to, or accessed through, an article. The authors have granted ASM a non-exclusive, world-wide license to publish the Supplemental Material files. Please contact the corresponding author directly for reuse.
