# Supplementary material for: Association between antimicrobial regimens and 28-day mortality in patients with Stenotrophomonas maltophilia infection: a propensity score-weighted analysis
Source: Microbiol Spectr. 2026 Jul 21;14(9):e01182-26. doi: 10.1128/spectrum.01182-26 (PMC13532249; doi:10.1128/spectrum.01182-26)
Supplement: Supplementary file 1 [file reviewer-comments.pdf]

AMERICAN SOCIETY FOR MICROBIOLOGY | Microbiology Spectrum

# Association Between Antimicrobial Regimens and 28-Day Mortality in Patients With *Stenotrophomonas maltophilia* Infection: A Propensity Score-Weighted Analysis

Shumin Gu, Xinyu Liu, Wenjing Lu, Shuzheng Du, Longhua Hu, Yunwei Zheng, xingwei cao, Yaping Hang, Zihan Li, Lihua Huang, and Qiaoshi Zhong

*Corresponding Author(s): Shumin Gu, the Second Affiliated Hospital of Nanchang University*

**Review Timeline:**

| | |
|---|---|
| Submission Date: | April 5, 2026 |
| Editorial Decision: | May 8, 2026 |
| Revision Received: | June 12, 2026 |
| Accepted: | June 15, 2026 |

*Editor: Andrea Prinzi*

*Reviewer(s): Disclosure of reviewer identity is with reference to reviewer comments included in decision letter(s). The following individuals involved in review of your submission have agreed to reveal their identity: Tristan Timbrook (Reviewer #1)*

## Transaction Report:

(Note: With the exception of the correction of typographical or spelling errors that could be a source of ambiguity, letters and reports are not edited. The original formatting of letters and referee reports may not be reflected in this compilation.)

**DOI: https://doi.org/10.1128/spectrum.01182-26**

Re: Spectrum01182-26 (**Comparative Effectiveness of Antimicrobial Regimens for *Stenotrophomonas maltophilia* Infections Based on a Propensity Score-Weighted Analysis**)

Dear Dr. Shumin Gu:

Thank you for the privilege of reviewing your work. Below you will find my comments, instructions from the Spectrum editorial office, and the reviewer comments.

Please return the manuscript within 60 days; if you cannot complete the modification within this time period, please contact me. If you do not wish to modify the manuscript and prefer to submit it to another journal, notify me immediately so that the manuscript may be formally withdrawn from consideration by Spectrum.

**Revision Guidelines**
To submit your modified manuscript, log into the submission site at https://spectrum.msubmit.net/cgi-bin/main.plex. Go to Author Tasks and click the appropriate manuscript title to begin. The information you entered when you first submitted the paper will be displayed; update this as necessary. Note the following requirements:

• Upload point-by-point responses to the issues raised by the reviewers in a file named "Response to Reviewers," NOT in your cover letter.
• Upload a compare copy of the manuscript (without figures) as a "Marked-Up Manuscript" file.
• Upload a clean .DOC/.DOCX version of the revised manuscript and remove the previous version.
• Each figure must be uploaded as a separate, editable, high-resolution file (TIFF or EPS preferred), and any multipanel figures must be assembled into one file.
• Any supplemental material intended for posting by ASM should be uploaded with their legends separate from the main manuscript. You can combine all supplemental material into one file (preferred) or split it into a maximum of 10 files with all associated legends included.

For complete guidelines on revision requirements, see our Submission and Review Process webpage. Submission of a paper that does not conform to guidelines may delay acceptance of your manuscript.

**Data availability:** ASM policy requires that data be available to the public upon online posting of the article, so please verify all links to sequence records, if present, and make sure that each number retrieves the full record of the data. If a new accession number is not linked or a link is broken, provide Spectrum production staff with the correct URL for the record. If the accession numbers for new data are not publicly accessible before the expected online posting of the article, publication may be delayed; please contact production staff (Spectrum@asmusa.org) immediately with the expected release date.

**Publication Fees:** For information on publication fees and which article types are subject to charges, visit our website. If your manuscript is accepted for publication and any fees apply, you will be contacted separately about payment during the production process; please follow the instructions in that e-mail. Arrangements for payment must be made before your article is published.

**ASM Membership:** Corresponding authors may join or renew ASM membership to obtain discounts on publication fees. Need to upgrade your membership level? Please contact Customer Service at Service@asmusa.org.

The ASM Journals program strives for constant improvement in our submission and publication process. Please tell us how we can improve your experience by taking this quick Author Survey.

Thank you for submitting your paper to Spectrum.

Sincerely,
Andrea Prinzi
Editor
Microbiology Spectrum

Reviewer #1 (Comments for the Author):

The current manuscript is a retrospective analysis of hospitalized adults with steno infections. The authors evaluate the comparison of various therapy modalities related to monotherapy and combination. They found combination therapy was associated with improved 28-day mortality. These findings are interesting and helpful given the limited outcomes literature for steno as indicated by the IDSA MDR guidelines. However, there are several areas of considerations for the authors before

accepting for publication.

Did the authors evaluate the role of potential role of resistance in confounding? Given the operational definition of definitive therapy as having in vitro activity, combination therapy patients by definition were more likely to have less likelihood of higher historical antibiotic exposures as well as related healthcare exposures and comorbidities. Given the definition, including resistance in IPTW would be a causality positivity violation. Thus would suggest the authors perform a sensitivity analysis of restricting to patients without in vitro resistance (or a compelling contraindication like allergy) to combination therapy in both arms. Would also discuss in limitations.

The authors use causal language throughout but use statistical significance to select potential confounders as well as present full models with beta-coefficients and p-values in tables 2&3. For prediction modeling, the approach proposed herein may be acceptable. But as noted by the American Thoracic Society (PMID: 30230362; and modern epidemiologists/biostatisticians in CONSORT explanation paper), stepwise selection is inappropriate for causal models and controlling confounding. Would revise for a causal model. Would omit tables 2&3 as reflect table 2 fallacy if considering as a causal model (PMID: 23371353). Else would revise language, conclusions as an association model. Depending on how the authors proceed, please select the appropriate reporting guideline from equator network, follow, and add to supplement (e.g. STROBE vs TRIPOD).

Given in nearly every cohort, some patients have multiple infections / infection sites, a pie graph would not be appropriate as it is consistent with a 100% total while the distribution of multiple infections per patient can lead to percentages greater than 100. Revise.

Ensure noting the limitation of cefoperazone-sulbactam based on regional resource limited use/availability, though potentially generalizable for other combination therapy.

Also please add limitation on lack of polymicrobial infections given steno often is in polymicrobial infections.

Reviewer #2 (Comments for the Author):

Summary

Gu et al. present a retrospective single-center cohort study evaluating antimicrobial treatment strategies for Stenotrophomonas maltophilia infections over a four-year period. Using IPTW, the authors examine the association between treatment regimen and 28-day all-cause mortality and report that combination therapy, particularly cefoperazone-sulbactam (CSL)-based regimens, was associated with improved survival compared with monotherapy. These findings may be relevant in resource-limited settings where access to newer antimicrobial agents is limited, although their generalizability is constrained in regions such as the United States and Europe where CSL is not routinely used. While the application of IPTW strengthens the analysis, several aspects of study design and reporting limit the strength and interpretability of the conclusions.

Introduction

It is stated that S. maltophilia's "intrinsic virulence is relatively low." This is not an accurate statement because S. maltophilia's intrinsic resistance mechanisms contribute to the difficulty of treatment and resistance to first-line beta-lactam therapies. Please amend.

IDSA-recommended treatments for S. maltophilia are mentioned, including cefiderocol, minocycline, TMP-SMX, and levofloxacin; however, the role of cefoperazone-sulbactam (CSL) is not introduced until the discussion despite being central to the study design. Additional background regarding CSL, including its general place in therapy for both S. maltophilia and other Gram-negative infections, should be included in the introduction and further expanded upon in the discussion to better orient readers, particularly those outside regions where CSL is routinely used. Additionally, ciprofloxacin and moxifloxacin are grouped with levofloxacin as fluoroquinolones despite not being considered standard-of-care agents for S. maltophilia. Their clinical role and limitations should also be discussed in the background section.

Materials and Methods

The authors should include additional time-to-event variables, including time from admission to culture collection, time from culture collection to organism identification, time to susceptibility results, and time to definitive therapy initiation. CSL performed well in both monotherapy and combination regimens and is commonly used for other Gram-negative infections. If CSL was initiated prior to identification of S. maltophilia, this may have shortened time to effective therapy and skewed results. The same concern applies to the other antibiotics that may have been initiated empirically for unrelated indications.

Patients with structural lung disease were not mentioned as exclusions. If these patients were included, this information should be incorporated into the baseline characteristics table. All exclusions should be listed in the methods according to figure 1. For example, patients with cystic fibrosis are mentioned in figure 1 as excluded, but this is not mentioned in the manuscript. Please update.

It is not specified whether patients who died early, transitioned to palliative care, or were transferred into or out of the hospital were excluded. For example, if a patient died or was transferred within 1-2 days of admission, were they still included in the analysis?
Please clarify the population included under "chronic respiratory failure." Were these patients receiving supplemental oxygen, and if so, what type (e.g., mechanical ventilation, BiPAP, nasal cannula)?

Duration of definitive therapy should be included, as there is no mention in the methods of a minimum duration of definitive therapy required for inclusion.

Regarding the definition of combination therapy, was there a specific rationale for selecting a 48-hour cutoff for concurrent therapy initiation? Please provide a breakdown of therapies initiated simultaneously versus therapies added within the 48-hour window. Addition of a second agent 48 hours later may represent escalation of therapy and should be acknowledged as such. Additionally, was there a minimum duration of overlap required for therapy to be considered combination therapy? For example, would a single overlapping dose qualify? How were patients categorized if they initially received one agent, later had a second agent added, and then completed therapy with a single agent?

Intracranial hemorrhage was included, which may have increased early non-infectious mortality in a subset of patients. Pulmonary hemorrhage, which may be more directly associated with severe S. maltophilia infection, was not included or discussed. The authors may consider excluding patients with intracranial hemorrhage who died early after admission and including pulmonary hemorrhage, if applicable.

Please elaborate on the definition of "onset of infection" being determined by the date of first positive culture. It is later stated that definitive therapy could be initiated up to 2 days before positive culture identification. Would therapy initiated before culture positivity not be considered empiric therapy?

Central venous catheters are not included among the relevant invasive procedures, although they are associated with S. maltophilia bacteremia. Please consider including this variable.

The definition of immunosuppression includes all patients with HIV; however, patients with undetectable viral loads and CD4 counts >200 cells/mcL are generally not considered immunosuppressed. Please address this in the discussion. Additionally, for patients who were post-transplant recipients or received chemotherapy or immunosuppressive medications, was there a defined temporal cutoff? For example, would a patient who underwent transplantation ten years prior but had not received immunosuppressive therapy for several years still meet criteria? Please clarify whether patients were actively immunosuppressed at the time of infection.

Diagnostic Criteria

Quantitative thresholds are provided for respiratory cultures to differentiate infection from colonization. Is quantitative respiratory culture routinely performed at this institution? The cited 2016 IDSA HAP/VAP guidelines note that quantitative cultures may be useful in possible VAP, but evidence supporting quantitative cultures in HAP is limited. Please clarify the patient population included in this analysis.
• Please provide citations supporting the clinical utility of quantitative cutoffs used for each respiratory specimen type further broken down by patients with HAP and VAP.

Quantitative thresholds are not provided for urinary cultures, raising the possibility that some included patients had asymptomatic bacteriuria. This should be addressed in the limitations section. Please elaborate.

Please clarify the statement that "other types of infection were defined according to CDC/NHSN criteria," as the citation associated with this sentence does not appear to correspond to CDC/NHSN guidance.

TMP-SMX monotherapy was not included in this study, despite being an important comparator for CSL/TMP-SMX combination therapy. Is there a hospital guideline or institutional practice pattern that prevented TMP-SMX monotherapy use, or were there other reasons for its exclusion?

Ciprofloxacin, moxifloxacin, and levofloxacin were all grouped together as fluoroquinolones, although ciprofloxacin has limited clinical efficacy against S. maltophilia because of resistance concerns, and moxifloxacin is generally limited to respiratory infections and may be cost-prohibitive. Levofloxacin is generally considered the preferred fluoroquinolone for S. maltophilia. Combining all fluoroquinolones into a single statistical variable may therefore skew results. Please elaborate on the rationale for this study design. Analyses should ideally be reported at the level of each individual fluoroquinolone.

Patients were required to receive definitive therapy for at least 70% of the treatment course; however, treatment duration itself was not defined. Please clarify.

For the definition of definitive therapy, does the "2 days before or after positive culture" refer to culture collection or organism identification?

Please provide citations supporting the dosing regimens used in this study.
• Levofloxacin 500 mg every 12 hours instead of 750 mg every 24 hours
• Clarify whether ciprofloxacin was administered intravenously only, as 400 mg every 8 hours corresponds to IV dosing
• TMP-SMX dosing is reported as 0.96 g every 8 or 12 hours. Please clarify whether this refers to 960 mg total daily dose divided every 8-12 hours or 960 mg per dose

Statistical Analysis

Regarding IPTW covariate selection:
• Covariate selection was reported to be based on variables differing significantly between groups at P < 0.05; however, only respiratory score, cardiovascular score, immunocompromised condition, and coma were listed. Immunocompromised condition and cardiovascular score do not appear statistically significant in Table 1.
• Variables that appear statistically significant include older age, ICU admission, invasive procedures, pulmonary infection, chronic respiratory failure, major bleeding, shock, neurological score, respiratory score, and coma.
• Please clarify or revise the covariate selection methodology or reconsider the variables included in the propensity score model.

Please include a power calculation based on prior literature.

Results

Microbiological Characteristics of S. maltophilia Isolates

Resistance rates to commonly used antibiotics are discussed; however, resistant isolates were excluded from the study. Please elaborate on the relevance and interpretation of these resistance data within the context of this analysis.

If the resistance figure is retained, please incorporate drugs without established S. maltophilia breakpoints and clearly note in the figure legend that surrogate Enterobacterales breakpoints were used where applicable. The figure should comprehensively address all included antimicrobials.

The manuscript inconsistently uses the terms "major bleeding" and "massive hemorrhage." Please standardize terminology throughout.

Discussion

The discussion raises concern regarding inadequate fluoroquinolone target attainment. Were the fluoroquinolone dosing regimens used in this study appropriate for pharmacodynamic target attainment? Additionally, because resistant isolates were excluded, pharmacodynamic targets should theoretically be achievable with appropriate therapeutic dosing.

Figures

Figure 3
The manuscript notes that there was no significant difference in resistance rates between pulmonary and non-pulmonary isolates; however, it is unclear whether statistical comparisons between antimicrobial agents themselves were performed. Please clarify.

Minocycline demonstrated the lowest resistance rates in both pulmonary and non-pulmonary isolates, yet its clinical role in this patient population is not discussed and minocycline-containing regimens were not included as comparator groups. Please provide rationale for this in both the introduction and discussion.

Supplementary Figure S4.
The manuscript states that all standardized mean differences after IPTW adjustment were <0.1; however, the cardiovascular systems score appears to remain >0.1. Please clarify.
Other

Consider defining abbreviations at first use and then using them consistently throughout the manuscript, as terminology currently alternates between abbreviated and non-abbreviated forms.

## Summary

Gu et al. present a retrospective single-center cohort study evaluating antimicrobial treatment strategies for *Stenotrophomonas maltophilia* infections over a four-year period. Using IPTW, the authors examine the association between treatment regimen and 28-day all-cause mortality and report that combination therapy, particularly cefoperazone-sulbactam (CSL)-based regimens, was associated with improved survival compared with monotherapy. These findings may be relevant in resource-limited settings where access to newer antimicrobial agents is limited, although their generalizability is constrained in regions such as the United States and Europe where CSL is not routinely used. While the application of IPTW strengthens the analysis, several aspects of study design and reporting limit the strength and interpretability of the conclusions.

## Introduction

It is stated that *S. maltophilia's* "intrinsic virulence is relatively low." This is not an accurate statement because *S. maltophilia's* intrinsic resistance mechanisms contribute to the difficulty of treatment and resistance to first-line beta-lactam therapies. Please amend.

IDSA-recommended treatments for *S. maltophilia* are mentioned, including cefiderocol, minocycline, TMP-SMX, and levofloxacin; however, the role of cefoperazone-sulbactam (CSL) is not introduced until the discussion despite being central to the study design. Additional background regarding CSL, including its general place in therapy for both *S. maltophilia* and other Gram-negative infections, should be included in the introduction and further expanded upon in the discussion to better orient readers, particularly those outside regions where CSL is routinely used. Additionally, ciprofloxacin and moxifloxacin are grouped with levofloxacin as fluoroquinolones despite not being considered standard-of-care agents for *S. maltophilia*. Their clinical role and limitations should also be discussed in the background section.

## Materials and Methods

The authors should include additional time-to-event variables, including time from admission to culture collection, time from culture collection to organism identification, time to susceptibility results, and time to definitive therapy initiation. CSL performed well in both monotherapy and combination regimens and is commonly used for other Gram-negative infections. If CSL was initiated prior to identification of *S. maltophilia*, this may have shortened time to effective therapy and skewed results. The same concern applies to the other antibiotics that may have been initiated empirically for unrelated indications.

Patients with structural lung disease were not mentioned as exclusions. If these patients were included, this information should be incorporated into the baseline characteristics table. All exclusions should be listed in the methods according to figure 1. For example, patients with cystic fibrosis are mentioned in figure 1 as excluded, but this is not mentioned in the manuscript. Please update.

It is not specified whether patients who died early, transitioned to palliative care, or were transferred into or out of the hospital were excluded. For example, if a patient died or was transferred within 1–2 days of admission, were they still included in the analysis?
Please clarify the population included under "chronic respiratory failure." Were these patients receiving supplemental oxygen, and if so, what type (e.g., mechanical ventilation, BiPAP, nasal cannula)?

Duration of definitive therapy should be included, as there is no mention in the methods of a minimum duration of definitive therapy required for inclusion.

Regarding the definition of combination therapy, was there a specific rationale for selecting a 48-hour cutoff for concurrent therapy initiation? Please provide a breakdown of therapies initiated simultaneously versus therapies added within the 48-hour window. Addition of a second agent 48 hours later may represent escalation of therapy and should be acknowledged as such. Additionally, was there a minimum duration of overlap required for therapy to be considered combination therapy? For example, would a single overlapping dose qualify? How were patients categorized if they initially received one agent, later had a second agent added, and then completed therapy with a single agent?

Intracranial hemorrhage was included, which may have increased early non-infectious mortality in a subset of patients. Pulmonary hemorrhage, which may be more directly associated with severe *S. maltophilia* infection, was not included or discussed. The authors may consider excluding patients with intracranial hemorrhage who died early after admission and including pulmonary hemorrhage, if applicable.

Please elaborate on the definition of "onset of infection" being determined by the date of first positive culture. It is later stated that definitive therapy could be initiated up to 2 days before positive culture identification. Would therapy initiated before culture positivity not be considered empiric therapy?

Central venous catheters are not included among the relevant invasive procedures, although they are associated with *S. maltophilia* bacteremia. Please consider including this variable.

The definition of immunosuppression includes all patients with HIV; however, patients with undetectable viral loads and CD4 counts >200 cells/mcL are generally not considered immunosuppressed. Please address this in the discussion. Additionally, for patients who were post-transplant recipients or received chemotherapy or immunosuppressive medications, was there a defined temporal cutoff? For example, would a patient who underwent transplantation ten years prior but had not received immunosuppressive therapy for several years still meet criteria? Please clarify whether patients were actively immunosuppressed at the time of infection.

*Diagnostic Criteria*

Quantitative thresholds are provided for respiratory cultures to differentiate infection from colonization. Is quantitative respiratory culture routinely performed at this institution? The cited 2016 IDSA HAP/VAP guidelines note that quantitative cultures may be useful in possible VAP,

but evidence supporting quantitative cultures in HAP is limited. Please clarify the patient population included in this analysis.

- Please provide citations supporting the clinical utility of quantitative cutoffs used for each respiratory specimen type further broken down by patients with HAP and VAP.

Quantitative thresholds are not provided for urinary cultures, raising the possibility that some included patients had asymptomatic bacteriuria. This should be addressed in the limitations section. Please elaborate.

Please clarify the statement that "other types of infection were defined according to CDC/NHSN criteria," as the citation associated with this sentence does not appear to correspond to CDC/NHSN guidance.

TMP-SMX monotherapy was not included in this study, despite being an important comparator for CSL/TMP-SMX combination therapy. Is there a hospital guideline or institutional practice pattern that prevented TMP-SMX monotherapy use, or were there other reasons for its exclusion?

Ciprofloxacin, moxifloxacin, and levofloxacin were all grouped together as fluoroquinolones, although ciprofloxacin has limited clinical efficacy against *S. maltophilia* because of resistance concerns, and moxifloxacin is generally limited to respiratory infections and may be cost-prohibitive. Levofloxacin is generally considered the preferred fluoroquinolone for *S. maltophilia*. Combining all fluoroquinolones into a single statistical variable may therefore skew results. Please elaborate on the rationale for this study design. Analyses should ideally be reported at the level of each individual fluoroquinolone.

Patients were required to receive definitive therapy for at least 70% of the treatment course; however, treatment duration itself was not defined. Please clarify.

For the definition of definitive therapy, does the "2 days before or after positive culture" refer to culture collection or organism identification?

Please provide citations supporting the dosing regimens used in this study.

- Levofloxacin 500 mg every 12 hours instead of 750 mg every 24 hours
- Clarify whether ciprofloxacin was administered intravenously only, as 400 mg every 8 hours corresponds to IV dosing
- TMP-SMX dosing is reported as 0.96 g every 8 or 12 hours. Please clarify whether this refers to 960 mg total daily dose divided every 8–12 hours or 960 mg per dose

*Statistical Analysis*

Regarding IPTW covariate selection:

- Covariate selection was reported to be based on variables differing significantly between groups at $P < 0.05$; however, only respiratory score, cardiovascular score, immunocompromised condition, and coma were listed. Immunocompromised condition and cardiovascular score do not appear statistically significant in Table 1.

- Variables that appear statistically significant include older age, ICU admission, invasive procedures, pulmonary infection, chronic respiratory failure, major bleeding, shock, neurological score, respiratory score, and coma.
- Please clarify or revise the covariate selection methodology or reconsider the variables included in the propensity score model.

Please include a power calculation based on prior literature.

Results

*Microbiological Characteristics of* S. maltophilia *Isolates*

Resistance rates to commonly used antibiotics are discussed; however, resistant isolates were excluded from the study. Please elaborate on the relevance and interpretation of these resistance data within the context of this analysis.

If the resistance figure is retained, please incorporate drugs without established *S. maltophilia* breakpoints and clearly note in the figure legend that surrogate Enterobacterales breakpoints were used where applicable. The figure should comprehensively address all included antimicrobials.

The manuscript inconsistently uses the terms "major bleeding" and "massive hemorrhage." Please standardize terminology throughout.

Discussion

The discussion raises concern regarding inadequate fluoroquinolone target attainment. Were the fluoroquinolone dosing regimens used in this study appropriate for pharmacodynamic target attainment? Additionally, because resistant isolates were excluded, pharmacodynamic targets should theoretically be achievable with appropriate therapeutic dosing.

Figures

*Figure 3*
The manuscript notes that there was no significant difference in resistance rates between pulmonary and non-pulmonary isolates; however, it is unclear whether statistical comparisons between antimicrobial agents themselves were performed. Please clarify.

Minocycline demonstrated the lowest resistance rates in both pulmonary and non-pulmonary isolates, yet its clinical role in this patient population is not discussed and minocycline-containing regimens were not included as comparator groups. Please provide rationale for this in both the introduction and discussion.

*Supplementary Figure S4.*
The manuscript states that all standardized mean differences after IPTW adjustment were <0.1; however, the cardiovascular systems score appears to remain >0.1. Please clarify.

Other

Consider defining abbreviations at first use and then using them consistently throughout the manuscript, as terminology currently alternates between abbreviated and non-abbreviated forms.

Reviewer #1 (Comments for the Author):

The current manuscript is a retrospective analysis of hospitalized adults with steno infections. The authors evaluate the comparison of various therapy modalities related to monotherapy and combination. They found combination therapy was associated with improved 28-day mortality. These findings are interesting and helpful given the limited outcomes literature for steno as indicated by the IDSA MDR guidelines. However, there are several areas of considerations for the authors before accepting for publication.

1.Did the authors evaluate the role of potential role of resistance in confounding? Given the operational definition of definitive therapy as having in vitro activity, combination therapy patients by definition were more likely to have less likelihood of higher historical antibiotic exposures as well as related healthcare exposures and comorbidities. Given the definition, including resistance in IPTW would be a causality positivity violation. Thus would suggest the authors perform a sensitivity analysis of restricting to patients without in vitro resistance (or a compelling contraindication like allergy) to combination therapy in both arms. Would also discuss in limitations.

**Response:** Thank you for this important comment. We agree that antimicrobial resistance profiles may have influenced treatment allocation and therefore could represent an important source of treatment-allocation bias.

In response to the reviewer's suggestion, we performed an additional resistance-restricted sensitivity analysis. For this analysis, eligibility was restricted to patients whose isolates had documented in vitro susceptibility to all three study drugs/classes—cefoperazone-sulbactam, fluoroquinolones, and trimethoprim-sulfamethoxazole—and who had no documented contraindications to these agents, such as drug allergy. After applying these criteria, 208 patients were

included in the resistance-restricted cohort, as shown in Supplementary Table S3, **which is presented below.**

Within this restricted cohort, propensity scores were re-estimated, IPTW weights were recalculated, covariate balance was reassessed, and weighted Cox proportional hazards models were refitted for both the comparison of combination therapy versus monotherapy and the four-regimen comparison. The results of this sensitivity analysis were generally consistent with the primary analysis. In the resistance-restricted cohort, combination therapy remained associated with lower 28-day all-cause mortality compared with monotherapy (HR = 0.236, 95% CI: 0.118–0.472, $P < 0.001$). In the regimen-specific analysis, FQ monotherapy continued to show the highest mortality estimate, whereas CSL plus TMP-SMX showed the lowest mortality estimate, although the reduced sample size and event numbers in the restricted cohort limited the precision of some regimen-specific comparisons.

We have added **the methods and results** of this resistance-restricted sensitivity analysis to the revised manuscript and **Supplementary Tables S3 and S5**. We have also revised the **Limitations section** to acknowledge that antimicrobial resistance profiles may have influenced treatment allocation and that, despite the resistance-restricted sensitivity analysis, residual confounding from prior antimicrobial exposure, healthcare exposure, prescribing preference, or undocumented contraindications cannot be excluded.

The revised text in the **Statistical Analysis** section now reads (Page 16, Lines 282-294): "To assess the robustness of findings to potential treatment-allocation bias related to antimicrobial resistance, we performed an additional resistance-restricted sensitivity analysis. Because the evaluated regimens consisted of FQ monotherapy, CSL monotherapy, CSL plus FQ, and CSL plus TMP-SMX, eligibility for this analysis was defined as documented in vitro susceptibility to all three study drugs/classes, namely CSL, FQ, and TMP-SMX, and absence of documented contraindications to these agents, such as drug allergy (Supplementary Table S6).

Within this restricted cohort, propensity scores were re-estimated, IPTW weights were recalculated, covariate balance was reassessed using standardized mean differences (Supplementary Figures S5), and weighted Cox proportional hazards models were refitted for both the comparison of combination therapy versus monotherapy and the four-regimen comparison (Supplementary Table S7)."

The revised text in the **Results** section now reads (Page 19, Lines 337-340 and Page 20-21, Lines 366-369): "Among the 208 patients included in the restricted cohort, combination therapy remained associated with lower 28-day all-cause mortality compared with monotherapy (HR = 0.236, 95% CI: 0.118-0.472, $P < 0.001$)."

"In the restricted cohort, the direction of regimen-specific estimates remained similar to the primary analysis, with FQ monotherapy showing the highest hazard estimate and CSL plus TMP-SMX showing the lowest hazard estimate."

The revised text in the **Limitations** section now reads (Page 26, Lines 464-470): "Second, antimicrobial resistance profiles may have influenced treatment allocation. Although a resistance-restricted sensitivity analysis was performed, residual confounding from prior antimicrobial exposure, healthcare exposure, prescribing preference, or undocumented contraindications cannot be excluded. In addition, the restricted cohort had fewer patients and events, particularly in the CSL plus TMP-SMX group, which may have limited the power of regimen-specific comparisons."

The newly added Supplementary Table S3 is presented below:

Supplementary Table S3. Antimicrobial Regimens and 28-Day Mortality in the Primary Cohort and Resistance-Restricted Sensitivity Analysis Cohort

| Treatment Approach | Regimen | Primary cohort, n | Primary cohort deaths, n/N (%) | Resistance-restricted cohort, n | Resistance-restricted cohort deaths, n/N (%) |
|---|---|---|---|---|---|
| Monotherapy | Cefoperazone-sulbactam | 58 | 16/58 (27.6%) | 43 | 12/43 (27.9%) |
| | Fluoroquinolone (levofloxacin, moxifloxacin, or ciprofloxacin) | 95 | 50/95 (52.6%) | 80 | 44/80 (55.0%) |
| Combination therapy | Cefoperazone-sulbactam plus fluoroquinolone | 63 | 12/63 (19.0%) | 50 | 9/50 (18.0%) |
| | Cefoperazone-sulbactam plus trimethoprim-sulfamethoxazole | 45 | 3/45 (6.7%) | 35 | 2/35 (5.7%) |
| Total | — | 261 | 81/261 (31.0%) | 208 | 67/208 (32.2%) |

**Note:** The resistance-restricted cohort included patients whose isolates were susceptible in vitro to cefoperazone-sulbactam, fluoroquinolones, and trimethoprim-sulfamethoxazole and who had no documented contraindications to these agents.

The newly added Supplementary Table S5 is presented below:

Supplementary Table S5. Sensitivity Analysis Comparing IPTW-Weighted Cox Regression Results in the Primary and Resistance-Restricted Cohorts

| **Analysis** | **Primary cohort HR (95% CI), *P* value** | **Resistance-restricted cohort HR (95% CI), *P* value** |
|---|---|---|
| Combination vs monotherapy | 0.235 (0.128–0.434), *P*<0.001 | 0.236 (0.118–0.472), *P*<0.001 |
| FQ vs CSL | 2.345 (1.325–4.149), *P*=0.003 | 2.344 (1.215–4.521), *P*=0.010 |
| CSL+FQ vs CSL | 0.572 (0.261–1.252), *P*=0.162 | 0.509 (0.206–1.258), *P*=0.143 |
| CSL+TMP-SMX vs CSL | 0.158 (0.038–0.653), *P*=0.010 | 0.204 (0.041–1.003), *P*=0.050 |

2.The authors use causal language throughout but use statistical significance to select potential confounders as well as present full models with beta-coefficients and p-values in tables 2&3. For prediction modeling, the approach proposed herein may be acceptable. But as noted by the American Thoracic Society (PMID: 30230362; and modern epidemiologists/biostatisticians in CONSORT explanation paper), stepwise selection is inappropriate for causal models and controlling confounding. Would revise for a causal model. Would omit tables 2&3 as reflect table 2 fallacy if considering as a causal model (PMID: 23371353). Else would revise language, conclusions as an association model. Depending on how the authors proceed, please select the appropriate reporting guideline from equator network, follow, and add to supplement (e.g. STROBE vs TRIPOD).

**Response:** Thank you for this important and insightful comment. We agree that, given the retrospective observational design, causal language should be avoided and

the analyses should be interpreted as association analyses rather than causal effect estimation.

Accordingly, we have revised the manuscript throughout, including the **Title, Abstract, Materials and Methods, Results, Discussion, and Conclusion**, to frame the study as an association model. Specifically, causal or prediction-oriented expressions such as “effect,” “independent predictors,” “risk factors,” “improved survival,” “beneficial,” and “performed better” were revised to association-based wording, such as “was associated with,” “showed lower hazard estimates,” “observed survival,” and “treatment-outcome associations.” We also revised the Statistical Analysis section to avoid presenting covariate selection as a stepwise causal-confounder selection process based solely on statistical significance. The propensity score model is now described as being based on clinically relevant baseline variables with observed imbalance across treatment groups.

We also agree with the concern regarding the potential Table 2 fallacy. Therefore, we have revised the table titles accordingly and clarified that Tables 2 and 3 should be interpreted as weighted Cox models evaluating associations with 28-day mortality, rather than as causal models estimating independent causal effects for each covariate. Because these revisions involved multiple sections of the manuscript, **the detailed textual changes are not exhaustively listed here but are marked in blue in the revised manuscript.**

Because this study is a retrospective observational cohort study evaluating associations between antimicrobial regimens and 28-day outcomes, we followed the STROBE reporting guideline. A completed STROBE checklist has been added as Supplementary File 2 and **is shown below**.

# STROBE Statement—Checklist of Items Included in Reports of Cohort Studies

*Completed checklist for the manuscript*

| **Section / Topic** | **Item No.** | **STROBE recommendation** | **Reported in revised manuscript** |
|---|---|---|---|
| **Title and abstract** | 1(a) | Indicate the study's design with a commonly used term in the title or the abstract. | **Page 2, Abstract—Methods**. The study design is identified as a retrospective cohort study: "We conducted a retrospective cohort study of adult inpatients with *S. maltophilia* infection between 2020 and 2023." |
| **Title and abstract** | 1(b) | Provide in the abstract an informative and balanced summary of what was done and what was found. | **Page 2–3, Abstract.** The abstract provides a structured summary of the study design, included patients, evaluated antimicrobial regimens, IPTW-weighted Cox and Kaplan-Meier analyses, 28-day all-cause mortality, main regimen-outcome associations, and cautious conclusions regarding the need for prospective multicenter validation. |
| **Introduction** | | | |
| Background/rationale | 2 | Explain the scientific background and rationale for the investigation being reported. | **Page 3–6, Introduction.** The scientific background and rationale are described, including the high mortality of *S. maltophilia* infection, limited therapeutic options, uncertainty regarding optimal therapy, and the need to evaluate commonly used regimens in real-world clinical practice. |
| Objectives | 3 | State specific objectives, including any prespecified hypotheses. | **Page 6, Introduction.** The study objective is stated at the end of the Introduction: to compare the association of commonly used therapeutic regimens, including FQ monotherapy, CSL monotherapy, CSL plus FQ, and CSL plus TMP-SMX, with 28-day outcomes in patients with *S. maltophilia* infection. |
| **Methods** | | | |
| Study design | 4 | Present key elements of study design early in the paper. | **Page 6, Materials and Methods—Study Design and Patient Population.** The retrospective cohort design, study setting, patient population, and study period are described at the beginning of the Methods section. |
| Setting | 5 | Describe the setting, locations, and relevant dates, including periods of recrui | **Page 6–8, Materials and Methods—Study Design and Patient Population;** |

| Section / Topic | Item No. | STROBE recommendation | Reported in revised manuscript |
|---|---|---|---|
| | | tment, exposure, follow-up, and data collection. | **Data collection.** The study setting, location, and relevant dates are describe d, including the single-center 3,600-bed tertiary hospital in southern China, th e study period from January 1, 2020 to December 31, 2023, the adult inpati ent population, data sources from the LIS and HIS, and the 28-day follow-u p period after onset of *S. maltophilia* infection. |
| Participants | 6(a) | Give the eligibility criteria, and the sources and methods of selection of parti cipants. Describe methods of follow-up. | **Page 6–8, Materials and Methods—Study Design and Patient Population; Data collection; Figure 1.** Eligibility criteria, exclusion criteria, data source s, and participant selection methods are described. Adult inpatients with *S. m altophilia* infection from January 1, 2020 to December 31, 2023 were screen ed, and the selection process is shown in Figure 1. Follow-up was based on 28-day survival status after onset of *S. maltophilia* infection obtained from t he medical record system. |
| Participants | 6(b) | For matched studies, give matching criteria and number of exposed and unex posed. | Not applicable. This study did not use a matched design. Propensity score-ba sed IPTW was applied rather than matching. |
| Variables | 7 | Clearly define all outcomes, exposures, predictors, potential confounders, and effect modifiers. Give diagnostic criteria, if applicable. | **Page 8–14, Materials and Methods—Data collection; Definitions; Diagnost ic criteria for *S. maltophilia* infection; Antimicrobial treatment; Statistical Analysis.** Outcomes, treatment exposures, covariates, and diagnostic criteria are defined. The primary outcome was 28-day all-cause mortality. Treatment exposures included monotherapy, combination therapy, and the four study-defi ned definitive antimicrobial regimens. Clinical covariates and potential confou nders, including comorbidities, infection type, SOFA scores, shock, coma, chr onic respiratory failure, and site-related invasive procedures, were collected a nd defined where applicable. Diagnostic criteria for pulmonary, urinary tract, and other *S. maltophilia* infections are described. |
| Data sources/measurement | 8 | For each variable of interest, give sources of data and details of methods of | **Page 7–13, Materials and Methods—Data collection; Definitions; Diagnost** |

| Section / Topic | Item No. | STROBE recommendation | Reported in revised manuscript |
|---|---|---|---|
| | | assessment. Describe comparability of assessment methods if there is more t han one group. | **ic criteria for *S. maltophilia* infection; Antimicrobial treatment.** Data sour ces and measurement methods are described. Clinical and microbiological dat a were extracted from the LIS and HIS. Definitions are provided for treatme nt exposure, infection onset, comorbidities, immunocompromised status, site-re lated invasive procedures, major bleeding, and 28-day all-cause mortality. Dia gnostic criteria for pulmonary, urinary tract, and other *S. maltophilia* infectio ns are described. Antimicrobial treatment classification, dosing, and susceptibi lity interpretation are also specified. The same data sources, definitions, and assessment methods were applied across treatment groups. |
| Bias | 9 | Describe any efforts to address potential sources of bias. | **Page 6–14 and 28–29, Materials and Methods—Study Design and Patient Population; Definitions; Antimicrobial treatment; Statistical Analysis; Dis cussion—Limitations.** Potential sources of bias were addressed by applying p redefined eligibility criteria, including only the first episode of *S. maltophilia* infection, excluding polymicrobial infections to reduce attribution bias, defini ng definitive therapy using prespecified timing and susceptibility criteria, usin g propensity score-based IPTW to improve balance in measured baseline char acteristics, examining and truncating extreme IPTW weights, and performing sensitivity analyses, including a resistance-restricted analysis. Remaining poten tial biases and residual confounding are discussed in the Limitations section. |
| Study size | 10 | Explain how the study size was arrived at. | **Page 6 and Page 14–15, Materials and Methods; Figure 1.** The study size was determined by the number of eligible patients identified during the retr ospective study period. The selection process is shown in Figure 1, and a po st hoc power calculation is described in the Statistical Analysis section. |
| Quantitative variables | 11 | Explain how quantitative variables were handled in the analyses. If applicabl e, describe which groupings were chosen and why. | **Page 13–14, Statistical Analysis; Table 1.** Quantitative or ordinal variables were categorized for analysis where applicable, including age ≥60 years and |

| Section / Topic | Item No. | STROBE recommendation | Reported in revised manuscript |
|---|---|---|---|
| | | | organ-specific SOFA scores ≥2. Categorical variables were summarized as fre quencies and percentages. |
| Statistical methods | 12(a) | Describe all statistical methods, including those used to control for confoundi ng. | **Page 13–15, Materials and Methods—Statistical Analysis.** Statistical metho ds are described, including descriptive analyses, chi-square tests, propensity s core estimation, IPTW to improve balance in measured baseline characteristic s across treatment groups, SMD-based balance assessment, weighted Cox pro portional hazards models, IPTW-adjusted Kaplan–Meier analyses, pairwise co mparisons with Tukey correction, and sensitivity analyses including resistance-restricted analyses. |
| Statistical methods | 12(b) | Describe any methods used to examine subgroups and interactions. | Not applicable. No formal subgroup or interaction analyses were performed. Sensitivity analyses are described in the Statistical Analysis section. |
| Statistical methods | 12(c) | Explain how missing data were addressed. | **Page 6, Materials and Methods—Study Design and Patient Population.** P atients with incomplete clinical, microbiological, treatment, or outcome data were excluded. No imputation was performed. |
| Statistical methods | 12(d) | If applicable, explain how loss to follow-up was addressed. | **Page 6–8, Materials and Methods.** Patients with incomplete outcome data w ere excluded. The 28-day survival status was obtained from the medical reco rd system, and no separate loss-to-follow-up analysis was applicable. |
| Statistical methods | 12(e) | Describe any sensitivity analyses. | **Page 13–15, Materials and Methods—Statistical Analysis.** Sensitivity analys es included refitting weighted Cox models using stabilized IPTW weights, un truncated IPTW weights, and a resistance-restricted cohort with re-estimated p ropensity scores and IPTW weights. |
| **Results** | | | |
| Participants | 13(a) | Report numbers of individuals at each stage of study—eligible, examined, co nfirmed eligible, included, completing follow-up, and analysed. | **Page 7 and Page 15, Figure 1; Results—Distribution of Infection Types.** The numbers of patients at each stage of screening and selection are shown in Figure 1. A total of 261 eligible patients were included in the final analy |

| Section / Topic | Item No. | STROBE recommendation | Reported in revised manuscript |
|---|---|---|---|
| | | | sis, and the Results section reports the final analysed cohort. |
| Participants | 13(b) | Give reasons for non-participation at each stage. | **Page 6–7, Materials and Methods—Study Design and Patient Population; Figure 1.** Reasons for exclusion are described in the eligibility criteria and shown in the patient selection flow chart, including age <18 years, polymicrobial infection, definitive antimicrobial therapy <48 hours, receipt of regimens other than the four study-defined regimens, cystic fibrosis, incomplete clinical/microbiological/treatment/outcome data, and in vitro resistance to the antibiotics used for definitive therapy. |
| Participants | 13(c) | Consider use of a flow diagram. | **Page 7, Figure 1.** A flow diagram describing patient screening, exclusions, and final inclusion is provided in Figure 1. |
| Descriptive data | 14(a) | Give characteristics of study participants and information on exposures and potential confounders. | **Page 16–18, Results—Demographic and Clinical Characteristics of the Study Population; Table 1.** Participant characteristics, treatment exposures, and potential confounders are reported in Table 1. |
| Descriptive data | 14(b) | Indicate number of participants with missing data for each variable of interest. | **Page 6, Materials and Methods—Study Design and Patient Population.** Patients with incomplete clinical, microbiological, treatment, or outcome data were excluded; therefore, no missing data were present for the main analysis variables in the final analytic cohort. |
| Descriptive data | 14(c) | Summarise follow-up time. | **Page 9, Materials and Methods—Definitions.** The follow-up period was 28 days from the onset of *S. maltophilia* infection, and 28-day all-cause mortality was assessed as the primary outcome. |
| Outcome data | 15 | Report numbers of outcome events or summary measures over time. | **Page 16, Results—Demographic and Clinical Characteristics; Figures 4 and 6.** The number of outcome events is reported as 81 deaths among 261 patients within 28 days. Kaplan–Meier curves summarize 28-day survival over time by treatment group. |
| Main results | 16(a) | Give unadjusted estimates and, if applicable, confounder-adjusted estimates a | **Page 16–24, Results; Tables 1–3; Figures 4–6; Page 13–15, Statistical Ana** |

| Section / Topic | Item No. | STROBE recommendation | Reported in revised manuscript |
|---|---|---|---|
| | | nd their precision. Make clear which confounders were adjusted for and why they were included. | **lysis.** Descriptive outcome data and treatment-group distributions are reported in the Results and Table 1. IPTW-weighted estimates with 95% CIs and P values are reported in Tables 2–3 and Figures 4–6 for the primary and regimen-specific analyses. Covariates included in the propensity score model and the rationale for their inclusion are described in the Statistical Analysis section. |
| Main results | 16(b) | Report category boundaries when continuous variables were categorized. | **Page 16–18, Table 1.** Category boundaries for categorized variables are reported, including age ≥60 years, hospitalization >30 days, and organ-specific SOFA scores ≥2. |
| Main results | 16(c) | If relevant, consider translating estimates of relative risk into absolute risk for a meaningful time period. | **Page 16–24, Results; Tables 2–3; Figures 4–6.** Hazard ratios with 95% CIs are reported, and absolute 28-day mortality/survival information is provided through mortality proportions and Kaplan–Meier survival curves. |
| Other analyses | 17 | Report other analyses done—eg analyses of subgroups and interactions, and sensitivity analyses. | **Page 19–24, Results; Supplementary Tables S4–S6; Supplementary Figure S5.** Sensitivity analyses are reported, including stabilized IPTW weights, untruncated IPTW weights, and a resistance-restricted cohort analysis. No formal subgroup or interaction analyses were performed. |
| **Discussion** | | | |
| Key results | 18 | Summarise key results with reference to study objectives. | **Page 24–28, Discussion.** Key findings are summarized with reference to the study objective, including 28-day mortality, treatment-outcome associations for monotherapy versus combination therapy, regimen-specific comparisons, and clinical factors associated with mortality. |
| Limitations | 19 | Discuss limitations of the study, taking into account sources of potential bias or imprecision. Discuss direction and magnitude of potential bias if appropriate. | **Page 28–29, Discussion—Limitations.** The limitations section discusses potential sources of bias and imprecision, including residual confounding, treatment-allocation bias related to antimicrobial resistance, reduced power in the restricted cohort, exclusion of polymicrobial infections, fluoroquinolone heterogenei |

| Section / Topic | Item No. | STROBE recommendation | Reported in revised manuscript |
|---|---|---|---|
| | | | ty, and limited generalizability. The direction and magnitude of these potentia l biases could not be precisely quantified. |
| Interpretation | 20 | Give a cautious overall interpretation considering objectives, limitations, mult iplicity of analyses, results from similar studies, and other relevant evidence. | **Page 24–30, Discussion and Conclusion.** The findings are interpreted cautio usly with reference to the study objectives, previous studies, guideline recom mendations, multiple regimen-specific comparisons, and study limitations. The need for prospective multicenter validation is stated. |
| Generalisability | 21 | Discuss the generalisability (external validity) of the study results. | **Page 28–30, Discussion—Limitations; Conclusion.** External validity is discus sed, including limitations related to the single-center design, exclusion of pol ymicrobial infections, region-specific CSL use, and restricted access to newer antimicrobial agents. |
| **Other information** | | | |
| Funding | 22 | Give the source of funding and the role of the funders. | **Page 30, Declarations—Funding.** The funding source and the role of the fu nder are reported. This work was supported by the Jiangxi Provincial Natural Science Foundation [grant number 20252BAC240578], and the funder had n o role in the study design, data collection, data analysis, data interpretation, manuscript preparation, or decision to submit the manuscript for publication. |

3.Given in nearly every cohort, some patients have multiple infections / infection sites, a pie graph would not be appropriate as it is consistent with a 100% total while the distribution of multiple infections per patient can lead to percentages greater than 100. Revise.

**Response:** Thank you for this helpful comment. We agree that a pie chart would be inappropriate if patients could be assigned to multiple infection-site categories, because the percentages could then exceed 100%. However, in this study, only the first episode of S. maltophilia infection was analyzed, and each included episode was assigned to one primary infection site for classification. Therefore, the percentages shown in Figure 2 were calculated from non-overlapping analytic categories and summed to 100%. We have clarified this point in the **figure legend**. The revised text now reads (Page 39, Lines 710-712): "Figure 2. Distribution of pulmonary and non-pulmonary infection types in patients with *S. maltophilia*. Each patient was classified according to a single primary infection-site category for analysis."

The underlying data for Figure 2 are shown below:

| **Infection site** | **No. of patients** |
|---|---|
| Pulmonary infections | 203 |
| Non-pulmonary infections | 58 |
| Total | 261 |

| **Non-pulmonary infection site** | **No. of patients** |
|---|---|
| Skin and soft tissue infections | 16 |
| Bloodstream infections | 13 |
| Urinary tract infections | 13 |
| Intra-abdominal infections | 8 |
| Biliary tract infections | 8 |
| Total | 58 |

4.Ensure noting the limitation of cefoperazone-sulbactam based on regional resource limited use/availability, though potentially generalizable for other combination therapy.

**Response:** Thank you for this helpful comment. We have revised the Limitations section to clarify that the applicability of CSL-based findings may be influenced by regional drug availability.

The revised text now reads (Page 27, Lines 479-483): "Finally, the non-evaluation of several guideline-recommended regimens due to local drug availability and institutional practice, together with the region-specific use of CSL, may limit the generalizability of our findings. Nevertheless, our findings reflect real-world care in a resource-limited setting and may inform treatment decisions in regions where access to newer antimicrobial agents is restricted."

5.Also please add limitation on lack of polymicrobial infections given steno often is in polymicrobial infections.

**Response:** Thank you for this helpful comment. We agree that exclusion of polymicrobial infections may limit the generalizability of our findings, because *S. maltophilia* is frequently recovered in polymicrobial infection settings. We excluded polymicrobial infections to reduce attribution bias when evaluating *S. maltophilia*-directed treatment effects.

We have added this limitation to the Discussion section as follows (Page 26, Lines 470-473): "Third, polymicrobial infections were excluded to reduce attribution bias in evaluating outcomes associated with *S. maltophilia*-directed treatment; however, because *S. maltophilia* is frequently recovered in polymicrobial infection settings, our findings may not be generalizable to polymicrobial infections."

Reviewer #2 (Comments for the Author):

**Summary**

Gu et al. present a retrospective single-center cohort study evaluating antimicrobial treatment strategies for Stenotrophomonas maltophilia infections over a four-year period. Using IPTW, the authors examine the association between treatment regimen and 28-day all-cause mortality and report that combination therapy, particularly cefoperazone-sulbactam (CSL)-based regimens, was associated with improved survival compared with monotherapy. These findings may be relevant in resource-limited settings where access to newer antimicrobial agents is limited, although their generalizability is constrained in regions such as the United States and Europe where CSL is not routinely used. While the application of IPTW strengthens the analysis, several aspects of study design and reporting limit the strength and interpretability of the conclusions.

**Introduction**

1.It is stated that S. maltophilia's "intrinsic virulence is relatively low." This is not an accurate statement because S. maltophilia's intrinsic resistance mechanisms contribute to the difficulty of treatment and resistance to first-line beta-lactam therapies. Please amend.

**Response:** Thank you for pointing this out. We agree that the statement “intrinsic virulence is relatively low” may be inaccurate and potentially misleading, particularly because the intrinsic resistance mechanisms of *S. maltophilia* contribute substantially to treatment difficulty and resistance to first-line β-lactam therapies. We have therefore removed this statement from the revised manuscript.

2.IDSA-recommended treatments for S. maltophilia are mentioned, including cefiderocol, minocycline, TMP-SMX, and levofloxacin; however, the role of cefop

erazone-sulbactam (CSL) is not introduced until the discussion despite being central to the study design. Additional background regarding CSL, including its general place in therapy for both S. maltophilia and other Gram-negative infections, should be included in the introduction and further expanded upon in the discussion to better orient readers, particularly those outside regions where CSL is routinely used.

**Response:** Thank you for this helpful comment. We agree that, because CSL-based regimens are central to the study design, additional background regarding CSL should be provided earlier in the manuscript to better orient readers, particularly those outside regions where CSL is routinely used.

We have therefore added background information on CSL in the **Introduction**, including its use as a β-lactam/β-lactamase inhibitor combination for Gram-negative infections in parts of Asia, its region-dependent availability and routine use, its limited role in current IDSA guidance for *S. maltophilia* infections, and the limited clinical evidence regarding CSL-based regimens for this pathogen. The revised text reads **(Page 6, Lines 98-109)**: "Cefoperazone-sulbactam (CSL), a β-lactam/β-lactamase inhibitor combination, is widely used in parts of Asia for Gram-negative infections, particularly in healthcare settings where access to newer antimicrobial agents is limited[15]. Although CSL is not a central agent in current IDSA guidance for *S. maltophilia* infections and its availability and routine use vary across regions, it remains clinically relevant in settings where it is readily available and commonly prescribed for multidrug-resistant Gram-negative infections[16]. Evidence regarding CSL-based regimens for *S. maltophilia* infections remains limited. Therefore, evaluating CSL monotherapy and CSL-based combination regimens in real-world clinical cohorts may provide useful clinical information for regions where CSL is part of routine antimicrobial practice."

We also expanded the **Discussion** to further address the clinical context of CSL-based regimens, including their potential relevance in resource-limited settings,

the limited evidence base for CSL in *S. maltophilia* infection, and the limitations in generalizability related to region-specific CSL use.

The revised text in the **Discussion** section now reads **(Page 24, Lines 426-430 and Page 27, Lines 476-483)**: "Although CSL is rarely used in Europe and the United States, it remains a pragmatic option in resource-limited Asian settings for multidrug-resistant Gram-negative infections[15]. Evidence for CSL monotherapy specifically against *S. maltophilia* has been limited; thus, our findings provide clinically relevant real-world data supporting its potential role."

"Finally, the non-evaluation of several guideline-recommended regimens due to local drug availability and institutional practice, together with the region-specific use of CSL, may limit the generalizability of our findings. Nevertheless, our findings reflect real-world care in a resource-limited setting and may inform treatment decisions in regions where access to newer antimicrobial agents is restricted."

3.Additionally, ciprofloxacin and moxifloxacin are grouped with levofloxacin as fluoroquinolones despite not being considered standard-of-care agents for S. maltophilia. Their clinical role and limitations should also be discussed in the background section.

**Response:** Thank you for this helpful comment. We agree that levofloxacin is generally the most commonly considered fluoroquinolone agent for *S. maltophilia*, whereas ciprofloxacin and moxifloxacin are not typically regarded as standard-of-care agents for this pathogen. We also agree that grouping these agents together as fluoroquinolones requires additional explanation and acknowledgment of its limitations.

Accordingly, we have revised the Introduction to clarify the clinical context of fluoroquinolone use. The revised text reads **(Page 6, Lines 92-98):** "Among fluoroquinolones, levofloxacin is generally the most commonly considered represe

ntative agent for *S. maltophilia*[12]. However, ciprofloxacin and moxifloxacin may also be encountered in selected patients in real-world clinical practice, and previous studies have evaluated or discussed these agents as fluoroquinolones in the context of in vitro activity or clinical relevance[13, 14]. Therefore, analyzing fluoroquinolone-containing regimens as a treatment category in retrospective real-world studies has a practical basis."

We have also expanded the Limitations section of the Discussion to acknowledge the limitation of analyzing fluoroquinolones as a single treatment category. The revised text reads **(Page 26, Lines 474-478):** "Fourth, FQs were analyzed as a single treatment category. Because levofloxacin, ciprofloxacin, and moxifloxacin may differ in antimicrobial activity, pharmacokinetic/pharmacodynamic properties, infection-site applicability, and supporting clinical evidence, this grouping may have introduced heterogeneity; however, the sample size was insufficient for robust analyses of individual FQs."

**Materials and Methods**

1.The authors should include **additional time-to-event variables**, including time from admission to culture collection, time from culture collection to organism identification, time to susceptibility results, and time to definitive therapy initiation. CSL performed well in both monotherapy and combination regimens and is commonly used for other Gram-negative infections. If CSL was initiated prior to identification of S. maltophilia, this may have shortened time to effective therapy and skewed results. The same concern applies to the other antibiotics that may have been initiated empirically for unrelated indications.

**Response:** Thank you for this important comment. We agree that time-related variables may influence the observed associations between antimicrobial regimens and outcomes, particularly if certain agents were initiated empirically before identification of *S. maltophilia* or before antimicrobial susceptibility results became available.

In response, we collected and compared additional time-related variables across the four treatment groups. These included time from hospital admission to first positive culture collection, time from culture collection to antimicrobial susceptibility reporting, days of use of any definitive-regimen agent before susceptibility reporting, and whether any definitive-regimen agent was initiated before susceptibility reporting.

At our institution, organism identification and antimicrobial susceptibility results are generally released together in the same microbiology report; therefore, time from culture collection to susceptibility reporting was used to represent the time to availability of both organism identification and susceptibility results. These data have been added to the **Data collection** section and summarized in **a new Supplementary Table S8**.

The results showed that time-related variables potentially affecting treatment timing were generally comparable across the four treatment groups. No statistically significant between-group differences were observed for time from hospital admission to first positive culture collection, days of use of any definitive-regimen agent before susceptibility reporting, or initiation of any definitive-regimen agent before susceptibility reporting. The time from culture collection to antimicrobial susceptibility reporting was borderline ($P = 0.050$), but the median values were similar across groups.

We have also added a brief statement in the **Results** section noting that these time-related variables were generally comparable across the four treatment groups.

The revised text in the **Data collection** section now reads **(Page 9, Lines 146-152):** "Clinical conditions at infection onset (arrhythmia, shock, hypoalbuminemia, coma), definitive antimicrobial treatment regimens, and time-related variables potentially affecting treatment timing were also collected. These time-related variables included time from hospital admission to first positive culture collection, time from culture collection to antimicrobial susceptibility reporting, days of use of any definitive-regimen agent before susceptibility reporting, and whethe

r any definitive-regimen agent was initiated before susceptibility reporting."

The revised text in the **Results** section now reads **(Page 20, Lines 348-352):**

"Building on the comparison between monotherapy and combination therapy, patients were further stratified into four antibiotic regimen groups (Supplementary Table S6). Time-related variables potentially affecting treatment timing were generally comparable across the four treatment groups (Supplementary Table S8)."

The newly added **Supplementary Table S8** is presented below.

Supplementary Table S8. Time-related variables across the four antimicrobial regimen groups

| **Time-related variable** | **CSL monotherapy** | **FQ monotherapy** | **CSL+ FQ** | **CSL+ TMP-SMX** | ***P* value** |
|---|---|---|---|---|---|
| Time from hospital admission to first positive culture collection, days | 12 (3–17) | 9 (3–17.5) | 13 (5–18.5) | 13 (4–22) | 0.486 |
| Time from culture collection to antimicrobial susceptibility reporting, days | 3 (2–3) | 2 (2–3) | 3 (2–3) | 3 (2–3) | 0.050 |
| Days of use of any definitive-regimen agent before susceptibility reporting | 2.5 (1–3) | 3 (2–3) | 3 (1–4) | 2 (1–3) | 0.167 |
| Any definitive-regimen agent initiated before susceptibility reporting, n/N (%) | 9/58 (15.5%) | 8/95 (8.4%) | 6/63 (9.5%) | 10/45 (22.2%) | 0.100 |

**Abbreviations:** CSL, cefoperazone-sulbactam; FQ, fluoroquinolone; TMP-SMX, trimethoprim-sulfamethoxazole.

**Note:** Continuous variables are presented as median (interquartile range). Categorical variables are presented as n/N (%). *P* values for continuous variables were

calculated using the Kruskal-Wallis test, and *P* values for categorical variables were calculated using the Pearson chi-square test.

2.Patients with structural lung disease were not mentioned as exclusions. If these patients were included, this information should be incorporated into the baseline characteristics table. All exclusions should be listed in the methods according to figure 1. For example, patients with cystic fibrosis are mentioned in figure 1 as excluded, but this is not mentioned in the manuscript. Please update.

**Response**: We thank the reviewer for this helpful comment. We have revised the **Methods** section to list all exclusion criteria according to Figure 1. We also clarified that patients with structural lung diseases other than cystic fibrosis were not excluded. Structural lung disease was additionally recorded and incorporated into **Table 1**. In this study, structural lung disease included chronic obstructive pulmonary disease, bronchiectasis, and interstitial lung disease/interstitial pneumonia documented in the medical record.

The revised text in the **Methods** section now reads **(Page 8, Lines 128-134 and Page 10, Lines 172-176):**

"Patients were excluded if they were younger than 18 years, had polymicrobial infections, received definitive antimicrobial therapy for less than 48 hours, received regimens other than the four study-defined regimens, had cystic fibrosis, had incomplete clinical, microbiological, treatment, or outcome data, or had isolates resistant in vitro to the antibiotics used for definitive therapy. Patients with structural lung diseases other than cystic fibrosis were not excluded. The detailed patient selection process is shown in Figure 1."

"Structural lung disease was defined as chronic pulmonary disease with documented structural abnormalities or chronic airway/lung remodeling in the medical record, including chronic obstructive pulmonary disease (COPD), bronchiectasis, and interstitial lung disease."

3.It is not specified whether patients who died early, transitioned to palliative care, or were transferred into or out of the hospital were excluded. For example, if a patient died or was transferred within 1-2 days of admission, were they still included in the analysis?

**Response**: We have revised the Methods section to list all exclusion criteria according to Figure 1. Patients who died or were transferred out before completing 48 hours of definitive antimicrobial therapy were classified as meeting the exclusion criterion of definitive antimicrobial therapy duration <48 hours. Patients whose 28-day survival status could not be ascertained were excluded as having incomplete outcome data. No patients transitioned to palliative care or were transferred in from another hospital in this cohort.

The revised text in the **Methods** section now reads **(Page 8, Lines 128-132):**

"Patients were excluded if they were younger than 18 years, had polymicrobial infections, received definitive antimicrobial therapy for less than 48 hours, received regimens other than the four study-defined regimens, had cystic fibrosis, had incomplete clinical, microbiological, treatment, or outcome data, or had isolates resistant in vitro to the antibiotics used for definitive therapy."

4.Please clarify the population included under "chronic respiratory failure." Were these patients receiving supplemental oxygen, and if so, what type (e.g., mechanical ventilation, BiPAP, nasal cannula)?

**Response:** We thank the reviewer for this helpful comment. We clarified the definition of chronic respiratory failure in the **Methods** section. Chronic respiratory failure was defined based on a documented pre-existing diagnosis before the onset of *S. maltophilia* infection, and baseline respiratory support was reviewed only to support this classification. Acute oxygen supplementation or mechanical ventilation initiated solely for the index infection was not considered chronic respiratory failure. Because the type of respiratory support was used to clarify the definition rather than as an analytical variable, it was not tabulated separately.

The revised text in the **Methods** section now reads **(Page 10, Lines 166-172):** "Chronic respiratory failure was defined as a documented diagnosis of chronic hypoxemic or hypercapnic respiratory failure before the onset of *S. maltophilia* infection. Baseline respiratory support, including oxygen therapy via nasal cannula or face mask, tracheostomy, or endotracheal intubation with invasive mechanical ventilation, was reviewed in the medical record. Acute oxygen supplementation or mechanical ventilation initiated solely for the index infection was not classified as chronic respiratory failure."

5.Duration of definitive therapy should be included, as there is no mention in the methods of a minimum duration of definitive therapy required for inclusion.

**Response:** We thank the reviewer for this helpful comment. We have revised the **Materials and methods** section to clarify the minimum duration of definitive antimicrobial therapy required for inclusion. Specifically, patients were required to receive definitive antimicrobial therapy for at least 48 hours and for at least 70% of the treatment course directed against *S. maltophilia*.

The revised text in the Materials and methods section now reads **(Page 13, Lines 235-237):** "Patients were required to receive definitive antimicrobial therapy for at least 48 hours and for at least 70% of the treatment course directed against *S. maltophilia*."

6.Regarding the definition of combination therapy, was there a specific rationale for selecting a 48-hour cutoff for concurrent therapy initiation? Please provide a breakdown of therapies initiated simultaneously versus therapies added within the 48-hour window. Addition of a second agent 48 hours later may represent escalation of therapy and should be acknowledged as such. Additionally, was there a minimum duration of overlap required for therapy to be considered combination therapy? For example, would a single overlapping dose qualify? How were patients categorized if they initially received one agent, later had a second agent added, and then completed therapy with a single agent?

**Response:** Thank you for this important comment. We agree that the rationale for the 48-hour window and the classification rules for combination therapy require clarification.

In this retrospective real-world study, the 48-hour cutoff was used as a pragmatic operational definition to capture early definitive combination therapy while allowing for routine delays in microbiological reporting, treatment modification, order entry, and drug administration. This time window is also consistent with prior infectious diseases studies that define timely or early active therapy as initiation within 48 hours of index culture collection[1].We have summarized the breakdown of simultaneous initiation versus addition of the second agent within the 48-hour window below.

| Combination regimen | Simultaneous initiation | Second agent added within 48 hours |
|---|---|---|
| CSL + FQ | 11/63 (17%) | 52/63 (83%) |
| CSL + TMP-SMX | 33/45 (73%) | 12/45 (27%) |
| Total | 44/108 (41%) | 64/108 (59%) |

We agree that later addition of a second active agent may represent treatment escalation rather than the initial definitive treatment strategy. In the present study, combination therapy was restricted to patients in whom two agents with documented in vitro activity against *S. maltophilia* were initiated concurrently or within 48 hours of each other. Patients in whom the second active agent was added more than 48 hours after the first agent were not classified as receiving combination therapy, because such changes were considered later treatment modification or escalation rather than the initial definitive regimen.

We have also clarified that a minimum overlap was required. To be classified as combination therapy, the two active agents had to overlap for at least 48 hours, and the overlapping two-agent regimen had to account for at least 70% of the definitive antimicrobial treatment course directed against *S. maltophilia*. A single overlapping dose or an overlap of less than 48 hours was not consider

ed combination therapy.

For patients who initially received one active agent, later had a second active agent added, and subsequently completed therapy with a single agent, classification was based on the predominant definitive regimen. If the combination regimen met the above criteria, the patient was classified as receiving combination therapy. If these criteria were not met but the predominant regimen fulfilled the definition of monotherapy, the patient was classified as receiving monotherapy. Patients whose treatment courses did not meet any of the four study-defined regimen categories were excluded.

We have revised the **Methods** section accordingly. Specifically, the revised text in the **Definitions** section now reads **(Page 9, Lines 157-161):** "Combination therapy was defined as the use of two agents with documented in vitro activity against the target pathogen, initiated concurrently or within 48 hours of each other and overlapping for at least 48 hours; patients in whom the second active agent was added more than 48 hours after initiation of the first active agent were not classified as receiving combination therapy."

The revised text in the **Antimicrobial treatment** section now reads **(Page 13, Lines 235-237):** "Patients were required to receive definitive antimicrobial therapy for at least 48 hours and for at least 70% of the treatment course directed against *S. maltophilia*."

7.Intracranial hemorrhage was included, which may have increased early non-infectious mortality in a subset of patients. Pulmonary hemorrhage, which may be more directly associated with severe S. maltophilia infection, was not included or discussed. The authors may consider excluding patients with intracranial hemorrhage who died early after admission and including pulmonary hemorrhage, if applicable.

**Response:** Thank you for this insightful comment. We agree that intracranial hemorrhage may contribute to early non-infectious mortality and could therefore influence estimates based on 28-day all-cause mortality.

We reviewed the non-survivors with intracranial hemorrhage to determine whether very early post-admission death related to intracranial bleeding may have affected the outcome analysis. Among the six non-survivors with intracranial hemorrhage, none died within 6 days after admission. The median interval from admission to first positive culture collection was 4 days (IQR, 3-10), the median interval from culture collection to death was 12 days (IQR, 5-14), and the median interval from admission to death was 17 days (IQR, 14-19). These findings suggest that these patients did not represent cases of very early post-admission death, and early non-infectious mortality solely attributable to intracranial hemorrhage was unlikely to materially drive the observed associations.

Regarding pulmonary hemorrhage, we reviewed the bleeding events recorded in the cohort. The major bleeding events identified in this study consisted of intracranial hemorrhage and gastrointestinal bleeding; no pulmonary hemorrhage meeting the major bleeding definition was identified.

| **Timing Characteristics Among Non-survivors With Intracranial Hemorrhage** | **Value** |
|---|---|
| Number of patients | 6 |
| Death within 6 days after admission, n (%) | 0/6 (0%) |
| Time from admission to first positive culture collection, days, median (IQR) | 4 (3-10) |
| Time from culture collection to death, days, median (IQR) | 12 (5-14) |
| Time from admission to death, days, median (IQR) | 17 (14-19) |

8.Please elaborate on the definition of "onset of infection" being determined by the date of first positive culture. It is later stated that definitive therapy could be initiated up to 2 days before positive culture identification. Would therapy initiated before culture positivity not be considered empiric therapy?

**Response:** Thank you for this helpful comment. We agree that the definition o

f infection onset and the distinction between empiric therapy at the time of ad ministration and retrospectively classified definitive therapy should be clarified. Because the exact clinical onset of infection could not be reliably determined i n this retrospective study, we used the date of collection of the first positive c ulture as an objective and reproducible operational index date for infection ons et. The revised text in the **Definitions** section now reads **(Page 9, Lines 162-1 64)**: "Because the exact clinical onset of infection could not be reliably determ ined retrospectively, the onset of *S. maltophilia* infection was operationally defi ned as the date of collection of the first positive culture."

We also agree that antimicrobial therapy initiated before culture positivity shoul d be considered empiric at the time of administration. However, for the purpos es of this retrospective analysis, such therapy was classified as definitive therap y if the agent was subsequently confirmed to have in vitro activity against the patient's *S. maltophilia* isolate and met the prespecified timing and duration c riteria. The revised text in the **Antimicrobial treatment** section now reads **(Pa ge 13, Lines 225-232)**: "Definitive therapy was defined as administration of ag ents to which the *S. maltophilia* isolate was susceptible in vitro and was initiat ed within 2 days before or up to 2 days after the date on which the first cult ure was reported positive for *S. maltophilia*. Antimicrobial therapy initiated bef ore culture positivity was considered empiric at the time of administration but was retrospectively classified as definitive therapy if the agent was subsequentl y confirmed to have in vitro activity against the patient's *S. maltophilia* isolate and met the prespecified timing and duration criteria."

9.Central venous catheters are not included among the relevant invasive proced ures, although they are associated with S. maltophilia bacteremia. Please consid er including this variable.

**Response:** Thank you for this helpful comment.We agree that central venous c atheterization is clinically relevant to *S. maltophilia* bloodstream infection. We r

e-reviewed the medical records of the 13 patients with bloodstream infection in our cohort.However, central venous catheterization was not documented in the available records for these patients. Therefore, this variable could not be reliably incorporated into the analysis.

10.The definition of immunosuppression includes all patients with HIV; however, patients with undetectable viral loads and CD4 counts >200 cells/mcL are generally not considered immunosuppressed. Please address this in the discussion. Additionally, for patients who were post-transplant recipients or received chemotherapy or immunosuppressive medications, was there a defined temporal cutoff? For example, would a patient who underwent transplantation ten years prior but had not received immunosuppressive therapy for several years still meet criteria? Please clarify whether patients were actively immunosuppressed at the time of infection.

**Response:** We thank the reviewer for this important comment. We agree that immunosuppression should reflect active immunosuppression at the onset of infection rather than remote history alone.

We reviewed the cohort and confirmed that no patients with HIV infection/AIDS or post-transplant status were identified; therefore, CD4 count, viral load, and remote transplantation status were not applicable to this cohort. In this study, immunosuppressive conditions related to cancer chemotherapy, systemic corticosteroids, biological immunomodulators, or other systemic immunosuppressive medications occurred during the index hospitalization and before or at the onset of *S. maltophilia* infection. We have revised the Methods to clarify that patients were considered immunosuppressed only when active immunosuppression was present before or at infection onset.

The revised text in the **Definitions** section now reads **(Page 11, Lines 186-191)**: "Patients were considered immunosuppressed if they had active immunosuppression during the index hospitalization and before or at the onset of *S. maltophilia* infection, including active cancer chemotherapy, systemic corticosteroid the

rapy equivalent to ≥20 mg prednisone per day for ≥14 days or a cumulative dose of ≥600 mg, biological immunomodulators, or other systemic immunosuppressive medications."

**Diagnostic Criteria**

1.Quantitative thresholds are provided for respiratory cultures to differentiate infection from colonization. Is quantitative respiratory culture routinely performed at this institution? The cited 2016 IDSA HAP/VAP guidelines note that quantitative cultures may be useful in possible VAP, but evidence supporting quantitative cultures in HAP is limited. Please clarify the patient population included in this analysis.

• Please provide citations supporting the clinical utility of quantitative cutoffs used for each respiratory specimen type further broken down by patients with HAP and VAP.

**Response:** We agree that the role of quantitative respiratory culture thresholds should be clarified, particularly because the evidence supporting quantitative cultures differs between HAP and VAP.

The pulmonary infection cohort included hospitalized adults with clinically diagnosed *S. maltophilia* pneumonia and was not restricted to patients with VAP. Therefore, we revised the diagnostic criteria to avoid applying quantitative culture thresholds uniformly as stand-alone criteria across all pneumonia cases.

We reviewed the microbiological workflow at our institution and clarified that quantitative culture was performed for bronchoalveolar lavage (BAL) specimens, whereas other respiratory specimens, including sputum and endotracheal aspirates, were generally reported semi-quantitatively. Therefore, respiratory culture results were interpreted according to specimen type and were not used as a stand-alone diagnostic criterion.

In the revised Methods, we clarified that pulmonary infections caused by *S. maltophilia* were diagnosed as pneumonia based on a combination of clinical, ra

diographic, oxygenation, and microbiological criteria. For BAL specimens, quantitative results were considered supportive microbiological evidence using a threshold of $10^4$ CFU/mL. For non-BAL respiratory specimens, semi-quantitative organism burden was considered supportive evidence and interpreted in conjunction with specimen quality and the overall clinical context. For sputum specimens, only qualified samples were considered. We also removed the implication that all respiratory cultures were interpreted solely according to quantitative thresholds.

The revised text in the **Diagnostic criteria for *S. maltophilia* infection** section now reads **(Page 11-12, Lines 195-212)**: "In this study, pulmonary infections caused by *S. maltophilia* were diagnosed as pneumonia based on a combination of clinical, radiographic, oxygenation, and microbiological criteria. At our institution, quantitative culture was performed for bronchoalveolar lavage (BAL) specimens, whereas other respiratory specimens were generally reported semi-quantitatively. Therefore, respiratory culture results were interpreted according to specimen type and were not used as a stand-alone diagnostic criterion. For BAL specimens, quantitative results were considered as supportive microbiological evidence, with a threshold of $10^4$ CFU/mL[18]. For non-BAL respiratory specimens, including sputum and endotracheal aspirates, semi-quantitative organism burden was considered as supportive evidence and interpreted in conjunction with specimen quality and the overall clinical context. For sputum specimens, only qualified samples were considered, defined as >25 neutrophils/LPF and <10 epithelial cells/LPF, or a neutrophil-to-epithelial cell ratio >2.5:1[19]. Pneumonia was further defined by the presence of a new or progressive pulmonary infiltrate on imaging, clinical findings such as fever (≥38 ℃) or hypothermia (≤36.5 ℃), leukocytosis (≥12,000/μL) or leukopenia (≤4,000/μL), purulent respiratory secretions, a positive respiratory specimen for *S. maltophilia*, and evidence of impaired oxygenation[20]."

2.Quantitative thresholds are not provided for urinary cultures, raising the possibility that some included patients had asymptomatic bacteriuria. This should be addressed in the limitations section. Please elaborate.

**Response:** We thank the reviewer for this helpful comment. We agree that urine culture positivity alone may not distinguish urinary tract infection from asymptomatic bacteriuria.

We reviewed the urinary infection cases and confirmed that all five patients met clinical criteria for urinary tract infection rather than asymptomatic bacteriuria, and that all five urinary isolates were recovered from midstream urine specimens.

We have revised the Methods to specify the quantitative threshold for urine culture and to clarify that clinical criteria were also required. The revised text in the **Diagnostic criteria for *S. maltophilia* infection** section now reads **(Page 12, Lines 212-219)**: "Urinary tract infection was defined as isolation of *S. maltophilia* from midstream urine at $\geqslant 10^5$ CFU/mL, in conjunction with compatible urinary symptoms, pyuria, fever or systemic signs of infection without a more likely alternative source[21], and clinician-documented diagnosis or treatment of urinary tract infection. Positive urine culture alone was not considered sufficient for the diagnosis of urinary tract infection, and asymptomatic bacteriuria was not classified as *S. maltophilia* infection."

3.Please clarify the statement that "other types of infection were defined according to CDC/NHSN criteria," as the citation associated with this sentence does not appear to correspond to CDC/NHSN guidance.

**Response:** Thank you for pointing this out. We apologize for the incorrect citation associated with this statement. We have corrected the reference and replaced it with the appropriate CDC/NHSN guidance.

The revised citation now reads **(Page 34, Lines 611-615)**: "[22] Centers for Disease Control and Prevention, National Healthcare Safety Network. CDC/NHSN surveillance definitions for specific types of infections. January 2026. Availabl

e from: https://www.cdc.gov/nhsn/pdfs/pscmanual/17pscnosinfdef_current.pdf. Accessed June 11, 2026."

4.TMP-SMX monotherapy was not included in this study, despite being an important comparator for CSL/TMP-SMX combination therapy. Is there a hospital guideline or institutional practice pattern that prevented TMP-SMX monotherapy use, or were there other reasons for its exclusion?

**Response:** We agree that TMP-SMX monotherapy would be an important comparator for CSL plus TMP-SMX combination therapy. However, among the 261 eligible patients in this real-world cohort, no patient received TMP-SMX monotherapy as definitive therapy. Therefore, TMP-SMX monotherapy was not excluded by study design but was absent from the observed treatment patterns during the study period. The specific reasons why TMP-SMX monotherapy was not used could not be reliably determined from the retrospective medical records. We have acknowledged in the Discussion that the regimens evaluated in this study were not comprehensive, which may limit the generalizability of our findings.

The revised text in the Discussion section now reads **(Page 27, Lines 479-481)**: "Finally, the non-evaluation of several guideline-recommended regimens due to local drug availability and institutional practice, together with the region-specific use of CSL, may limit the generalizability of our findings."

5.Ciprofloxacin, moxifloxacin, and levofloxacin were all grouped together as fluoroquinolones, although ciprofloxacin has limited clinical efficacy against S. maltophilia because of resistance concerns, and moxifloxacin is generally limited to respiratory infections and may be cost-prohibitive. Levofloxacin is generally considered the preferred fluoroquinolone for S. maltophilia. Combining all fluoroquinolones into a single statistical variable may therefore skew results. Please elaborate on the rationale for this study design. Analyses should ideally be reported at the level of each individual fluoroquinolone.

**Response:** Thank you for this important comment. We agree that individual fluoroquinolones may differ in antimicrobial activity, pharmacokinetic/pharmacodynamic properties, infection-site applicability, and supporting clinical evidence for *S. maltophilia* infection.

In this retrospective real-world study, fluoroquinolones were analyzed as a treatment category primarily because of sample size limitations. After stratification by individual agents, the numbers of patients receiving levofloxacin, ciprofloxacin, or moxifloxacin were too small to support robust adjusted or IPTW-weighted outcome analyses, and such estimates would be statistically unstable. In addition, fluoroquinolones have been analyzed as a class in some prior observational studies of *S. maltophilia* infection, which provided a practical basis for this treatment-category approach[2].

We acknowledge that grouping different fluoroquinolones together may have introduced within-class heterogeneity. We have revised the Limitations section to clarify this issue as follows **(Page 26, Lines 474-478)**: "Fourth, FQs were analyzed as a single treatment category. Because levofloxacin, ciprofloxacin, and moxifloxacin may differ in antimicrobial activity, pharmacokinetic/pharmacodynamic properties, infection-site applicability, and supporting clinical evidence, this grouping may have introduced heterogeneity; however, the sample size was insufficient for robust analyses of individual FQs."

6.Patients were required to receive definitive therapy for at least 70% of the treatment course; however, treatment duration itself was not defined. Please clarify.

**Response:** Thank you for this helpful comment. We agree that the denominator used for the 70% criterion should be clarified.

We have revised the **Antimicrobial treatment** section to define the treatment course as the duration of antimicrobial therapy directed against *S. maltophilia*. The revised text now reads **(Page 13, Lines 232-237)**: "The treatment course was defined as the duration of antimicrobial therapy directed against *S. maltophil*

*ia*, from initiation of the first agent subsequently confirmed to have in vitro activity against the isolate to discontinuation of anti-*S. maltophilia* therapy, death, or hospital discharge, whichever occurred first. Patients were required to receive definitive antimicrobial therapy for at least 48 hours and for at least 70% of the treatment course directed against *S. maltophilia*."

7.For the definition of definitive therapy, does the "2 days before or after positive culture" refer to culture collection or organism identification?

**Response:** Thank you for this helpful comment. We agree that the reference point for the 2-day window should be clarified.

The 2-day window referred to the date on which the first culture was reported positive for *S. maltophilia*. We have revised the **Antimicrobial treatment** section to clarify this point.

The revised text now reads **(Page 13, Lines 225-228)**: "Definitive therapy was defined as administration of agents to which the *S. maltophilia* isolate was susceptible in vitro and was initiated within 2 days before or up to 2 days after the date on which the first culture was reported positive for *S. maltophilia*."

8.Please provide citations supporting the dosing regimens used in this study.

- Levofloxacin 500 mg every 12 hours instead of 750 mg every 24 hours
- Clarify whether ciprofloxacin was administered intravenously only, as 400 mg every 8 hours corresponds to IV dosing
- TMP-SMX dosing is reported as 0.96 g every 8 or 12 hours. Please clarify whether this refers to 960 mg total daily dose divided every 8-12 hours or 960 mg per dose

**Response:** Thank you for this helpful comment. We agree that the dosing regimens should be described more clearly.

Because this was a retrospective real-world study, antimicrobial doses were not protocol-mandated by the investigators. Rather, the reported regimens reflected physician-prescribed treatment in routine clinical practice and were adjusted ac

cording to local drug labeling, renal function, infection severity, infection site, and individual patient tolerance. We have revised the Methods to clarify the route of administration and dose expression. Levofloxacin was administered at 500 mg every 12 hours according to local drug labeling and routine clinical practice. Ciprofloxacin was administered intravenously at 400 mg every 8 hours. Except for TMP-SMX, which was administered orally, the study antibiotics were administered intravenously. TMP-SMX 0.96 g referred to 0.96 g per dose of the fixed-dose combination, corresponding to trimethoprim 160 mg plus sulfamethoxazole 800 mg per dose, administered every 8 or 12 hours. Dose adjustment was performed based on renal function when clinically indicated.

The revised text now reads **(Page 13-14, Lines 237-247)**: "The dosing regimens reflected physician-prescribed treatment in routine clinical practice and were adjusted according to local drug labeling, renal function, infection severity, infection site, and individual patient tolerance. Except for TMP-SMX, which was administered orally, the study antibiotics were administered intravenously. The dosing regimens during treatment were as follows: levofloxacin 500 mg every 12 hours, ciprofloxacin 400 mg every 8 hours, moxifloxacin 400 mg every 24 hours, and CSL most commonly at 9 g per 24 hours in three divided doses, with less frequent use of 6 or 12 g per 24 hours. TMP-SMX was administered orally as 0.96 g per dose every 8 or 12 hours, corresponding to trimethoprim 160 mg plus sulfamethoxazole 800 mg per dose, with dose adjustment based on renal function when clinically indicated."

**Statistical Analysis**

1.Regarding IPTW covariate selection:

• Covariate selection was reported to be based on variables differing significantly between groups at $P < 0.05$; however, only respiratory score, cardiovascular score, immunocompromised condition, and coma were listed. Immunocompromised condition and cardiovascular score do not appear statistically significant in

Table 1.

- Variables that appear statistically significant include older age, ICU admission, invasive procedures, pulmonary infection, chronic respiratory failure, major bleeding, shock, neurological score, respiratory score, and coma.
- Please clarify or revise the covariate selection methodology or reconsider the variables included in the propensity score model.

**Response:** Thank you for this important comment. We agree that the description of covariate selection for the IPTW analysis should be clarified.

Table 1 in the manuscript summarizes baseline characteristics according to 28-day survival status, not according to treatment group. Therefore, the *P* values in Table 1 were not used to select covariates for the propensity score model. For IPTW construction, propensity scores were estimated using treatment assignment as the dependent variable. Covariates were selected based on baseline comparisons across treatment groups, rather than survival-status comparisons.

Specifically, the variables included in the propensity score model—respiratory system SOFA score ≥2, cardiovascular system SOFA score ≥2, immunocompromised condition, and coma—were those that differed significantly across treatment groups in the treatment-group baseline comparison. We have added this treatment-group baseline comparison as Supplementary Table S1 to make the covariate selection process transparent. We have revised the **Statistical Analysis** section to clarify this point.

The revised text now reads **(Page 14-15, Lines 257-263)**: "To reduce measured baseline confounding, propensity scores were estimated using a multinomial logistic regression model with treatment group as the dependent variable. Covariates for the propensity score model were selected based on clinically relevant baseline variables with observed imbalance across treatment groups, as shown in Supplementary Table S1. The propensity score model included respiratory system SOFA score ≥2, cardiovascular system SOFA score ≥2, immunocompromised condition, and coma."

The treatment-group baseline comparison is presented in Supplementary Table S1, as shown below:

Supplementary Table S1. Baseline characteristics of patients with *S. maltophilia* infection according to antimicrobial regimen

| Variables, n (%) | CSL N=58 | FQ N=95 | CSL plus FQ N=63 | CSL plus TMP-SMX N=45 | *P* value |
|---|---|---|---|---|---|
| **Older age (≥ 60 years)** | 34(58.6) | 60(63.2) | 37(58.7) | 22(48.9) | 0.464 |
| **Sex, male** | 39(67.2) | 72(75.8) | 48(76.2) | 29(64.4) | 0.369 |
| **Hospitalization for > 30 days prior to infection** | 22(37.9) | 34(35.8) | 27(42.9) | 22(48.9) | 0.477 |
| **ICU admission** | 21(36.2) | 31(32.6) | 33(52.4) | 22(48.9) | 0. 050 |
| **Parenteral nutrition** | 18(31.0) | 22(23.2) | 17(27.0) | 12(26.7) | 0.761 |
| **Surgical operation** | 13(22.4) | 21(22.1) | 21(33.3) | 8(17.8) | 0.239 |
| **Hemodialysis** | 7(12.1) | 8(8.4) | 8(12.7) | 5(11.1) | 0.826 |
| **Invasive procedures related to the site of infection** | 31(53.4) | 49(51.6) | 42(66.7) | 22(48.9) | 0.199 |
| **Pulmonary infection** | 46(79.3) | 71(74.7) | 47(74.6) | 39(86.7) | 0.390 |
| **Co-morbidities** | | | | | |
| Diabetes mellitus | 13(22.4) | 20(21.1) | 8(12.7) | 11(24.4) | 0.401 |
| Hypertension | 24(41.4) | 42(44.2) | 21(33.3) | 16(35.6) | 0.519 |
| Cerebrovascular disease | 17(29.3) | 18(18.9) | 12(19.0) | 10(22.2) | 0.448 |
| Chronic hepatic disease | 18(31.0) | 22(23.2) | 22(34.9) | 16(35.6) | 0.315 |
| Cardiovascular disease | 20(34.5) | 23(24.2) | 23(36.5) | 15(33.3) | 0.336 |
| Solid tumor | 14(24.1) | 25(26.3) | 11(17.5) | 6(13.3) | 0.268 |
| Hematologic malignancy | 4(6.9) | 3(3.2) | 4(6.3) | 4(8.9) | 0.549 |
| Chronic heart failure | 17(29.3) | 21(22.1) | 16(25.4) | 11(24.4) | 0.798 |
| Chronic respiratory failure | 28(48.3) | 42(44.2) | 28(44.4) | 27(60.0) | 0.326 |

| | | | | | |
|---|---|---|---|---|---|
| Chronic renal failure | 14(24.1) | 26(27.4) | 20(31.7) | 19(42.2) | 0.210 |
| Major bleeding | 8(13.8) | 16(16.8) | 12(19.0) | 13(28.9) | 0.241 |
| Immunocompromised condition | 9(15.5) | 29(30.5) | 21(33.3) | 23(51.1) | 0.002 |
| **Diagnosis at the onset of *S. maltophilia* infection** | | | | | |
| Arrhythmia | 15(25.9) | 17(18.0) | 13(20.6) | 10(22.2) | 0.701 |
| Shock | 11(19.0) | 15(16.0) | 11(17.5) | 12(26.7) | 0.481 |
| Hypoalbuminemia | 46(79.3) | 75(78.9) | 49(77.8) | 41(91.1) | 0.289 |
| Coma | 6(10.3) | 19(20.0) | 12(19.0) | 16(35.6) | 0.018 |
| **SOFA scoring** | | | | | |
| Respiratory systems Score ≥ 2 | 28(48.3) | 43(45.3) | 40(63.5) | 29(64.4) | 0.0476 |
| Cardiovascular systems Score ≥ 2 | 9(15.5) | 7(7.4) | 15(23.8) | 7(15.6) | 0.0391 |
| Neurological systems Score ≥ 2 | 15(25.9) | 32(33.7) | 22(34.9) | 19(42.2) | 0.377 |
| Liver system Score ≥ 2 | 7(12.1) | 14(14.7) | 11(17.5) | 3(6.7) | 0.410 |
| Kidney system Score ≥ 2 | 13(22.4) | 16(16.8) | 16(25.4) | 9(20.0) | 0.609 |
| Coagulation system Score ≥ 2 | 11(19.0) | 21(22.1) | 12(19.0) | 15(33.3) | 0.276 |

Abbreviations: CSL, cefoperazone-sulbactam; FQ, fluoroquinolone; TMP-SMX, trimethoprim-sulfamethoxazole; SOFA, Sequential Organ Failure Assessment.

Notes: P values represent overall comparisons across the four treatment groups. No pairwise post-hoc comparisons were performed.

2.Please include a power calculation based on prior literature.

**Response:** In response to the reviewer's suggestion, we performed a post hoc power calculation for the primary comparison between monotherapy and combination therapy. Based on prior studies reporting short-term mortality of approximately 40% among patients with *S. maltophilia* infection[3][4], we assumed a mortality rate of 40% in the monotherapy group and 25% in the combination-therapy group, corresponding to a clinically meaningful absolute mortality reduction of 15%. With 153 patients in the monotherapy group and 108 patients in the combination-therapy group, the estimated statistical power was approximately 73% at a two-sided α level of 0.05. This information has been added to the Statistical Analysis section.

The revised text now reads **(Page 16-17, Lines 294-299)**: "Given the retrospective design, a post hoc power calculation was performed for the primary comparison between monotherapy and combination therapy based on short-term mortality estimates reported in prior studies of *S. maltophilia* infection[25, 26]. Assuming mortality rates of 40% for monotherapy and 25% for combination therapy, the available sample size provided an estimated power of approximately 73% at a two-sided α level of 0.05."

**Results**

**Microbiological Characteristics of S. maltophilia Isolates**

1.Resistance rates to commonly used antibiotics are discussed; however, resistant isolates were excluded from the study. Please elaborate on the relevance and interpretation of these resistance data within the context of this analysis.

If the resistance figure is retained, please incorporate drugs without established *S. maltophilia* breakpoints and clearly note in the figure legend that surrogate Enterobacterales breakpoints were used where applicable. The figure should comprehensively address all included antimicrobials.

**Response:** Thank you for this important comment. We agree that the resistance data were descriptive and were not central to the comparative effectiveness analysis, because patients with isolates resistant to the antibiotics actually used for definitive therapy were excluded. To avoid overinterpretation and potential confusion regarding the relevance of resistance rates to the treatment-effect estimates, we have **removed the "Microbiological Characteristics of *S. maltophilia* Isolates" subsection and Figure 3** from the revised manuscript. We have retained the susceptibility interpretation methods in the Antimicrobial treatment section because documented in vitro activity was required for classification of definitive therapy.

2.The manuscript inconsistently uses the terms "major bleeding" and "massive hemorrhage." Please standardize terminology throughout.

**Response:** Thank you for pointing out this inconsistency. We have revised the manuscript to standardize the terminology and now use "major bleeding" consistently throughout the text and tables.

**Discussion**

1.The discussion raises concern regarding inadequate fluoroquinolone target attainment. Were the fluoroquinolone dosing regimens used in this study appropriate for pharmacodynamic target attainment? Additionally, because resistant isolates were excluded, pharmacodynamic targets should theoretically be achievable with appropriate therapeutic dosing.

**Response:** Thank you for this important comment. We agree that fluoroquinolone pharmacodynamic target attainment cannot be directly inferred in this retrospective study.

The fluoroquinolone regimens used in this cohort reflected physician-prescribed therapeutic dosing in routine clinical practice, and fluoroquinolone-treated patients had isolates with documented in vitro susceptibility to the administered agent

because resistant isolates were excluded. However, susceptibility alone does not ensure pharmacodynamic target attainment in individual patients, particularly in critically ill patients with altered pharmacokinetics.

Because MIC distributions, drug exposure data, and AUC/MIC target-attainment analyses were not available, we could not directly determine whether the fluoroquinolone regimens achieved pharmacodynamic targets. We have therefore revised the **Discussion** to avoid overinterpreting inadequate pharmacodynamic exposure as a definitive explanation for the poorer outcomes observed with FQ monotherapy. In the revised manuscript, inadequate pharmacodynamic exposure is discussed only as a possible contributing factor, rather than a confirmed mechanism.

The revised text now reads **(Page 24-25, Lines 433-444)**: "In contrast, FQs require adequate exposure (e.g., AUC/MIC) to achieve efficacy[37]. Although all FQ-treated patients in this study had isolates with documented in vitro susceptibility to the administered agent, susceptibility alone does not ensure pharmacodynamic target attainment in individual patients. In critically ill patients, exposure variability and safety-limited dose escalation may predispose to suboptimal target attainment[38] and resistance selection[5]. These considerations are consistent with the caution against levofloxacin monotherapy in severe *S. maltophilia* infections noted by CLSI[39]. However, because MIC distributions, drug exposure data, and AUC/MIC target-attainment analyses were not available, inadequate pharmacodynamic exposure can only be considered a possible contributing factor, rather than a confirmed explanation, for the poorer outcomes observed with FQ monotherapy."

**Figures**

**1.Figure 3**

1.1 The manuscript notes that there was no significant difference in resistance rates between pulmonary and non-pulmonary isolates; however, it is unclear wh

ether statistical comparisons between antimicrobial agents themselves were performed. Please clarify.

**Response:** Thank you for this helpful comment. Because Figure 3 and the corresponding resistance-rate analysis have been removed from the revised manuscript, the statistical comparisons shown in that figure are no longer applicable.

1.2 Minocycline demonstrated the lowest resistance rates in both pulmonary and non-pulmonary isolates, yet its clinical role in this patient population is not discussed and minocycline-containing regimens were not included as comparator groups. Please provide rationale for this in both the introduction and discussion.

**Response:** Thank you for this important comment. We agree that minocycline is an important potential therapeutic option for *S. maltophilia* infection.

However, in this cohort, minocycline monotherapy or minocycline-containing regimens were not used, or were used too infrequently, as definitive therapy in routine clinical practice at our institution. Therefore, these regimens could not form a meaningful comparator group and were not included in the comparative effectiveness analysis. We have clarified this limitation in the **Discussion** section, noting that several guideline-recommended regimens were not evaluated because of local drug availability and institutional practice, which may limit the generalizability of our findings.

The revised text in the **Discussion** section now reads **(Page 27, Lines 479-481)**: "Finally, the non-evaluation of several guideline-recommended regimens due to local drug availability and institutional practice, together with the region-specific use of CSL, may limit the generalizability of our findings."

**2.Supplementary Figure S4.**

2.1The manuscript states that all standardized mean differences after IPTW adjustment were <0.1; however, the cardiovascular systems score appears to remain >0.1. Please clarify.

**Response:** Thank you for pointing this out. We re-examined Supplementary Figure S4 and the underlying SMD values. In the four-regimen IPTW analysis using truncated weights, most post-weighting SMDs were <0.1. However, cardiovascular system SOFA score ≥2 showed a slight residual imbalance, with a maximum pairwise SMD of 0.107. We have revised the **Results** section to avoid stating that all post-weighting SMDs were <0.1 and to clarify this residual imbalance.

The revised text now reads **(Page 20, Lines 352-355)**:

"IPTW was then re-applied for the four-regimen analysis. Covariate balance was substantially improved after weighting. Most post-weighting SMDs were <0.1, although cardiovascular system SOFA score ≥2 remained marginally above the prespecified threshold (Supplementary Figure S4)."

**Other**

Consider defining abbreviations at first use and then using them consistently throughout the manuscript, as terminology currently alternates between abbreviated and non-abbreviated forms.

**Response:** Thank you for this helpful comment. We have carefully reviewed the manuscript and revised the terminology to ensure that abbreviations are defined at first use and then used consistently throughout the manuscript, tables, figure legends, and supplementary materials where applicable.

## References

[1] Kunz Coyne AJ, Lucas K, Gray R, May E. Propensity-matched comparison of timely vs delayed antibiotic therapy in *Stenotrophomonas maltophilia* pneumonia. Open Forum Infect Dis. 2025;12(8):ofaf469. https://doi.org/10.1093/ofid/ofaf469

[2] Junco SJ, Bowman MC, Turner RB. Clinical outcomes of *Stenotrophomonas maltophilia* infection treated with trimethoprim/sulfamethoxazole, minocyc

line, or fluoroquinolone monotherapy. Int J Antimicrob Agents. 2021;58(2): 106367. https://doi.org/10.1016/j.ijantimicag.2021.106367

[3] Chen L, Zhang L, Li X, Li B, He B, Liu B, et al. Assessment of the relative benefits of monotherapy and combination therapy approaches to the treatment of hospital-acquired *Stenotrophomonas maltophilia* pneumonia: a multicenter, observational, real-world study. Ann Intensive Care. 2023;13:77. https://doi.org/10.1186/s13613-023-01144-7

[4] Pongdumbun T, Rongrungruang Y, Boonyasiri A. P-1358. Efficacy of combination therapy with minocycline for the treatment of *Stenotrophomonas maltophilia* infections [abstract]. Open Forum Infect Dis. 2026;13(Suppl 1):ofaf695.1545. https://doi.org/10.1093/ofid/ofaf695.1545

Re: Spectrum01182-26R1 (**Association Between Antimicrobial Regimens and 28-Day Mortality in Patients With *Stenotrophomonas maltophilia* Infection: A Propensity Score-Weighted Analysis**)

Dear Dr. Shumin Gu:

Thank you for comprehensively responding to all reviewer feedback and questions. The author responses to the reviewer comments are thorough, rigorous, and appropriately address all concerns.

Your manuscript has been accepted, and I am forwarding it to the ASM production staff for publication. Your paper will first be checked to make sure all elements meet the technical requirements. ASM staff will contact you if anything needs to be revised before copyediting and production can begin. Otherwise, you will be notified when your proofs are ready to be viewed.

**Data Availability:** ASM policy requires that data be available to the public upon online posting of the article, so please verify all links to sequence records, if present, and make sure that each number retrieves the full record of the data. If a new accession number is not linked or a link is broken, provide production staff with the correct URL for the record. If the accession numbers for new data are not publicly accessible before the expected online posting of the article, publication may be delayed; please contact ASM production staff immediately with the expected release date.

**Publication Fees:** For information on publication fees and which article types have charges, please visit ourwebsite. We have partnered with Copyright Clearance Center (CCC) to collect author charges. If fees apply to your paper, you will receive a message from no-reply@copyright.com with further instructions. For questions related to paying charges through RightsLink, please contact CCC at ASM_Support@copyright.com or toll free at +1-877-622-5543. CCC makes every attempt to respond to all emails within 24 hours.

**ASM Membership:** Corresponding authors may join or renew ASM membership to obtain discounts on publication fees. Need to upgrade your membership level? Please contact Customer Service at Service@asmusa.org.

**Embargo Policy:** A press release may be issued as soon as the manuscript is posted on theSpectrum Latest Articles webpage. The corresponding author will receive an email with the subject line "ASM Journals Author Services Notification" when the article is available online.

The ASM Journals program strives for constant improvement in our submission and publication process. Please tell us how we can improve your experience by taking this quick Author Survey.

Thank you for submitting your paper to Spectrum.

Sincerely,
Andrea Prinzi
Editor
Microbiology Spectrum
