# Supplementary material for: Association between antimicrobial regimens and 28-day mortality in patients with Stenotrophomonas maltophilia infection: a propensity score-weighted analysis
Source: Microbiol Spectr. 2026 Jul 21;14(9):e01182-26. doi: 10.1128/spectrum.01182-26 (PMC13532249; doi:10.1128/spectrum.01182-26)
Supplement: Supplementary file 2 [file spectrum.01182-26-s0001.pdf]

# STROBE Statement—Checklist of Items Included in Reports of Cohort Studies

*Completed checklist for the manuscript*

| Section / Topic | Item No. | STROBE recommendation | Reported in revised manuscript |
|---|---|---|---|
| **Title and abstract** | 1(a) | Indicate the study's design with a commonly used term in the title or the abstract. | **Page 2, Abstract—Methods**. The study design is identified as a retrospective cohort study: "We conducted a retrospective cohort study of adult inpatients with *S. maltophilia* infection between 2020 and 2023." |
| **Title and abstract** | 1(b) | Provide in the abstract an informative and balanced summary of what was done and what was found. | **Page 2–3, Abstract.** The abstract provides a structured summary of the study design, included patients, evaluated antimicrobial regimens, IPTW-weighted Cox and Kaplan-Meier analyses, 28-day all-cause mortality, main regimen-outcome associations, and cautious conclusions regarding the need for prospective multicenter validation. |
| **Introduction** | | | |
| Background/rationale | 2 | Explain the scientific background and rationale for the investigation being reported. | **Page 3–6, Introduction.** The scientific background and rationale are described, including the high mortality of *S. maltophilia* infection, limited therapeutic options, uncertainty regarding optimal therapy, and the need to evaluate commonly used regimens in real-world clinical practice. |
| Objectives | 3 | State specific objectives, including any prespecified hypotheses. | **Page 6, Introduction.** The study objective is stated at the end of the Introduction: to compare the association of commonly used therapeutic regimens, including FQ monotherapy, CSL monotherapy, CSL plus FQ, and CSL plus TMP-SMX, with 28-day outcomes in patients with *S. maltophilia* infection. |
| **Methods** | | | |
| Study design | 4 | Present key elements of study design early in the paper. | **Page 6, Materials and Methods—Study Design and Patient Population.** The retrospective cohort design, study setting, patient population, and study period are described at the beginning of the Methods section. |
| Setting | 5 | Describe the setting, locations, and relevant dates, including periods of recruitment, exposure, follow-up, and data collection. | **Page 6–8, Materials and Methods—Study Design and Patient Population; Data collection.** The study setting, location, and relevant dates are described, including the single-center 3,600-bed tertiary hospital in southern China, the study period from January 1, 2020 to December 31, 2023, the adult inpatient population, data sources from the LIS and HIS, and the 28-day follow-up period after onset of *S. maltophilia* infection. |
| Participants | 6(a) | Give the eligibility criteria, and the sources and methods of selection of participants. Describe methods of follow-up. | **Page 6–8, Materials and Methods—Study Design and Patient Population; Data collection; Figure 1.** Eligibility criteria, exclusion criteria, data sources, and participant selection methods are described. Adult inpatients with *S. maltophilia* infection from January 1, 2020 to December 31, 2023 were screened, and the selection process is shown in Figure 1. Follow-up was based on 28-day survival status after onset of *S. maltophilia* infection obtained from the medical record system. |
| Participants | 6(b) | For matched studies, give matching criteria and number of exposed and unexposed. | Not applicable. This study did not use a matched design. Propensity score-based IPTW was applied rather than matching. |
| Variables | 7 | Clearly define all outcomes, exposures, predictors, potential confounders, and effect modifiers. Give diagnostic criteria, if applicable. | **Page 8–14, Materials and Methods—Data collection; Definitions; Diagnostic criteria for *S. maltophilia* infection; Antimicrobial treatment; Statistical Analysis.** Outcomes, treatment exposures, covariates, and diagnostic criteria are defined. The primary outcome was 28-day all-cause mortality. Treatment exposures included monotherapy, combination therapy, and the four study-defined definitive antimicrobial regimens. Clinical covariates and potential confounders, including comorbidities, infection type, SOFA scores, shock, |

| Section / Topic | Item No. | STROBE recommendation | Reported in revised manuscript |
|---|---|---|---|
| | | | coma, chronic respiratory failure, and site-related invasive procedures, were collected and defined where applicable. Diagnostic criteria for pulmonary, urinary tract, and other *S. maltophilia* infections are described. |
| Data sources/measurement | 8 | For each variable of interest, give sources of data and details of methods of assessment. Describe comparability of assessment methods if there is more than one group. | **Page 7–13, Materials and Methods—Data collection; Definitions; Diagnostic criteria for *S. maltophilia* infection; Antimicrobial treatment.** Data sources and measurement methods are described. Clinical and microbiological data were extracted from the LIS and HIS. Definitions are provided for treatment exposure, infection onset, comorbidities, immunocompromised status, site-related invasive procedures, major bleeding, and 28-day all-cause mortality. Diagnostic criteria for pulmonary, urinary tract, and other *S. maltophilia* infections are described. Antimicrobial treatment classification, dosing, and susceptibility interpretation are also specified. The same data sources, definitions, and assessment methods were applied across treatment groups. |
| Bias | 9 | Describe any efforts to address potential sources of bias. | **Page 6–14 and 28–29, Materials and Methods—Study Design and Patient Population; Definitions; Antimicrobial treatment; Statistical Analysis; Discussion—Limitations.** Potential sources of bias were addressed by applying predefined eligibility criteria, including only the first episode of *S. maltophilia* infection, excluding polymicrobial infections to reduce attribution bias, defining definitive therapy using prespecified timing and susceptibility criteria, using propensity score-based IPTW to improve balance in measured baseline characteristics, examining and truncating extreme IPTW weights, and performing sensitivity analyses, including a resistance-restricted analysis. Remaining potential biases and residual confounding are discussed in the Limitations section. |
| Study size | 10 | Explain how the study size was arrived at. | **Page 6 and Page 14–15, Materials and Methods; Figure 1.** The study size was determined by the number of eligible patients identified during the retrospective study period. The selection process is shown in Figure 1, and a post hoc power calculation is described in the Statistical Analysis section. |
| Quantitative variables | 11 | Explain how quantitative variables were handled in the analyses. If applicable, describe which groupings were chosen and why. | **Page 13–14, Statistical Analysis; Table 1.** Quantitative or ordinal variables were categorized for analysis where applicable, including age ≥60 years and organ-specific SOFA scores ≥2. Categorical variables were summarized as frequencies and percentages. |
| Statistical methods | 12(a) | Describe all statistical methods, including those used to control for confounding. | **Page 13–15, Materials and Methods—Statistical Analysis.** Statistical methods are described, including descriptive analyses, chi-square tests, propensity score estimation, IPTW to improve balance in measured baseline characteristics across treatment groups, SMD-based balance assessment, weighted Cox proportional hazards models, IPTW-adjusted Kaplan–Meier analyses, pairwise comparisons with Tukey correction, and sensitivity analyses including resistance-restricted analyses. |
| Statistical methods | 12(b) | Describe any methods used to examine subgroups and interactions. | Not applicable. No formal subgroup or interaction analyses were performed. Sensitivity analyses are described in the Statistical Analysis section. |
| Statistical methods | 12(c) | Explain how missing data were addressed. | **Page 6, Materials and Methods—Study Design and Patient Population.** Patients with incomplete clinical, microbiological, treatment, or outcome data were excluded. No imputation was performed. |
| Statistical methods | 12(d) | If applicable, explain how loss to follow-up was addressed. | **Page 6–8, Materials and Methods.** Patients with incomplete outcome data were excluded. The 28-day survival status was obtained from the medical record system, and no separate |

| Section / Topic | Item No. | STROBE recommendation | Reported in revised manuscript |
|---|---|---|---|
| | | | loss-to-follow-up analysis was applicable. |
| Statistical methods | 12(e) | Describe any sensitivity analyses. | **Page 13–15, Materials and Methods—Statistical Analysis.** Sensitivity analyses included refitting weighted Cox models using stabilized IPTW weights, untruncated IPTW weights, and a resistance-restricted cohort with re-estimated propensity scores and IPTW weights. |
| **Results** | | | |
| Participants | 13(a) | Report numbers of individuals at each stage of study—eligible, examined, confirmed eligible, included, completing follow-up, and analysed. | **Page 7 and Page 15, Figure 1; Results—Distribution of Infection Types.** The numbers of patients at each stage of screening and selection are shown in Figure 1. A total of 261 eligible patients were included in the final analysis, and the Results section reports the final analysed cohort. |
| Participants | 13(b) | Give reasons for non-participation at each stage. | **Page 6–7, Materials and Methods—Study Design and Patient Population; Figure 1.** Reasons for exclusion are described in the eligibility criteria and shown in the patient selection flow chart, including age <18 years, polymicrobial infection, definitive antimicrobial therapy <48 hours, receipt of regimens other than the four study-defined regimens, cystic fibrosis, incomplete clinical/microbiological/treatment/outcome data, and in vitro resistance to the antibiotics used for definitive therapy. |
| Participants | 13(c) | Consider use of a flow diagram. | **Page 7, Figure 1.** A flow diagram describing patient screening, exclusions, and final inclusion is provided in Figure 1. |
| Descriptive data | 14(a) | Give characteristics of study participants and information on exposures and potential confounders. | **Page 16–18, Results—Demographic and Clinical Characteristics of the Study Population; Table 1.** Participant characteristics, treatment exposures, and potential confounders are reported in Table 1. |
| Descriptive data | 14(b) | Indicate number of participants with missing data for each variable of interest. | **Page 6, Materials and Methods—Study Design and Patient Population.** Patients with incomplete clinical, microbiological, treatment, or outcome data were excluded; therefore, no missing data were present for the main analysis variables in the final analytic cohort. |
| Descriptive data | 14(c) | Summarise follow-up time. | **Page 9, Materials and Methods—Definitions.** The follow-up period was 28 days from the onset of *S. maltophilia* infection, and 28-day all-cause mortality was assessed as the primary outcome. |
| Outcome data | 15 | Report numbers of outcome events or summary measures over time. | **Page 16, Results—Demographic and Clinical Characteristics; Figures 4 and 6.** The number of outcome events is reported as 81 deaths among 261 patients within 28 days. Kaplan–Meier curves summarize 28-day survival over time by treatment group. |
| Main results | 16(a) | Give unadjusted estimates and, if applicable, confounder-adjusted estimates and their precision. Make clear which confounders were adjusted for and why they were included. | **Page 16–24, Results; Tables 1–3; Figures 4–6; Page 13–15, Statistical Analysis.** Descriptive outcome data and treatment-group distributions are reported in the Results and Table 1. IPTW-weighted estimates with 95% CIs and P values are reported in Tables 2–3 and Figures 4–6 for the primary and regimen-specific analyses. Covariates included in the propensity score model and the rationale for their inclusion are described in the Statistical Analysis section. |
| Main results | 16(b) | Report category boundaries when continuous variables were categorized. | **Page 16–18, Table 1.** Category boundaries for categorized variables are reported, including age ≥60 years, hospitalization >30 days, and organ-specific SOFA scores ≥2. |

| Section / Topic | Item No. | STROBE recommendation | Reported in revised manuscript |
|---|---|---|---|
| Main results | 16(c) | If relevant, consider translating estimates of relative risk into absolute risk for a meaningful time period. | **Page 16–24, Results; Tables 2–3; Figures 4–6.** Hazard ratios with 95% CIs are reported, and absolute 28-day mortality/survival information is provided through mortality proportions and Kaplan–Meier survival curves. |
| Other analyses | 17 | Report other analyses done—eg analyses of subgroups and interactions, and sensitivity analyses. | **Page 19–24, Results; Supplementary Tables S4–S6; Supplementary Figure S5.** Sensitivity analyses are reported, including stabilized IPTW weights, untruncated IPTW weights, and a resistance-restricted cohort analysis. No formal subgroup or interaction analyses were performed. |
| **Discussion** | | | |
| Key results | 18 | Summarise key results with reference to study objectives. | **Page 24–28, Discussion.** Key findings are summarized with reference to the study objective, including 28-day mortality, treatment-outcome associations for monotherapy versus combination therapy, regimen-specific comparisons, and clinical factors associated with mortality. |
| Limitations | 19 | Discuss limitations of the study, taking into account sources of potential bias or imprecision. Discuss direction and magnitude of potential bias if appropriate. | **Page 28–29, Discussion—Limitations.** The limitations section discusses potential sources of bias and imprecision, including residual confounding, treatment-allocation bias related to antimicrobial resistance, reduced power in the restricted cohort, exclusion of polymicrobial infections, fluoroquinolone heterogeneity, and limited generalizability. The direction and magnitude of these potential biases could not be precisely quantified. |
| Interpretation | 20 | Give a cautious overall interpretation considering objectives, limitations, multiplicity of analyses, results from similar studies, and other relevant evidence. | **Page 24–30, Discussion and Conclusion.** The findings are interpreted cautiously with reference to the study objectives, previous studies, guideline recommendations, multiple regimen-specific comparisons, and study limitations. The need for prospective multicenter validation is stated. |
| Generalisability | 21 | Discuss the generalisability (external validity) of the study results. | **Page 28–30, Discussion—Limitations; Conclusion.** External validity is discussed, including limitations related to the single-center design, exclusion of polymicrobial infections, region-specific CSL use, and restricted access to newer antimicrobial agents. |
| **Other information** | | | |
| Funding | 22 | Give the source of funding and the role of the funders. | **Page 30, Declarations—Funding.** The funding source and the role of the funder are reported. This work was supported by the Jiangxi Provincial Natural Science Foundation [grant number 20252BAC240578], and the funder had no role in the study design, data collection, data analysis, data interpretation, manuscript preparation, or decision to submit the manuscript for publication. |

***Note:*** *The original STROBE checklist for cohort studies was downloaded from the official STROBE website and completed according to the revised manuscript.*
