# Supplementary material for: Association between antimicrobial regimens and 28-day mortality in patients with Stenotrophomonas maltophilia infection: a propensity score-weighted analysis
Source: Microbiol Spectr. 2026 Jul 21;14(9):e01182-26. doi: 10.1128/spectrum.01182-26 (PMC13532249; doi:10.1128/spectrum.01182-26)
Supplement: Supplementary file 3 [file spectrum.01182-26-s0007.pdf]

Supplementary materials

Title: Comparative Effectiveness of Antimicrobial Regimens for *Stenotrophomonas maltophilia* Infections Based on a Propensity Score-Weighted Analysis

Journal:Infection

Authors: Shumin Gu[a†], Xinyu Liu[b†], Wenjing Lu[a], Shuzheng Du[c], Longhua Hu[a], Yunwei Zheng[a], Xingwei Cao[a], Yaping Hang[a], Zihan Li[a], Lihua Huang[a], Qiaoshi Zhong[a*]

Affiliations：[a]Department of Clinical Laboratory, Jiangxi Province Key Laboratory of Immunology and Inflammation, Jiangxi Provincial Clinical Research Center for Laboratory Medicine, The Second Affiliated Hospital, Jiangxi Medical College, Nanchang University, Nanchang, 330000, China.

[b]School of Clinical Medicine, Jiangxi University of Chinese Medicine, Nanchang, 330000, China.

[c]College of Anesthesiology, Henan Medical University，Xinxiang, 453000, China

[*]**Corresponding author:** Qiaoshi Zhong

Department of Clinical Laboratory, The Second Affiliated Hospital of Nanchang University, Mingde Road No. 1, Nanchang 330006, China

Email: zhong20000947@sina.com

[†]These authors contributed equally to this article.

Supplementary Table S1. Baseline characteristics of patients with *S. maltophilia* infection according to antimicrobial regimen

**Variables, n (%)**	**CSL**<br>**N=58**	**FQ**<br>**N=95**	**CSL plus FQ**<br>**N=63**	**CSL plus TMP-SMX**<br>**N=45**	***P* value**
**Older age (≥ 60 years)**	34(58.6)	60(63.2)	37(58.7)	22(48.9)	0.464
**Sex, male**	39(67.2)	72(75.8)	48(76.2)	29(64.4)	0.369
**Hospitalization for > 30 days prior to infection**	22(37.9)	34(35.8)	27(42.9)	22(48.9)	0.477
**ICU admission**	21(36.2)	31(32.6)	33(52.4)	22(48.9)	0.050
**Parenteral nutrition**	18(31.0)	22(23.2)	17(27.0)	12(26.7)	0.761
**Surgical operation**	13(22.4)	21(22.1)	21(33.3)	8(17.8)	0.239
**Hemodialysis**	7(12.1)	8(8.4)	8(12.7)	5(11.1)	0.826
**Invasive procedures related to the site of infection**	31(53.4)	49(51.6)	42(66.7)	22(48.9)	0.199
**Pulmonary infection**	46(79.3)	71(74.7)	47(74.6)	39(86.7)	0.390
**Co-morbidities**					

Diabetes mellitus	13(22.4)	20(21.1)	8(12.7)	11(24.4)	0.401
Hypertension	24(41.4)	42(44.2)	21(33.3)	16(35.6)	0.519
Cerebrovascular disease	17(29.3)	18(18.9)	12(19.0)	10(22.2)	0.448
Chronic hepatic disease	18(31.0)	22(23.2)	22(34.9)	16(35.6)	0.315
Cardiovascular disease	20(34.5)	23(24.2)	23(36.5)	15(33.3)	0.336
Solid tumor	14(24.1)	25(26.3)	11(17.5)	6(13.3)	0.268
Hematologic malignancy	4(6.9)	3(3.2)	4(6.3)	4(8.9)	0.549
Chronic heart failure	17(29.3)	21(22.1)	16(25.4)	11(24.4)	0.798
Chronic respiratory failure	28(48.3)	42(44.2)	28(44.4)	27(60.0)	0.326
Chronic renal failure	14(24.1)	26(27.4)	20(31.7)	19(42.2)	0.210
Major bleeding	8(13.8)	16(16.8)	12(19.0)	13(28.9)	0.241
Immunocompromised condition	9(15.5)	29(30.5)	21(33.3)	23(51.1)	0.002
**Diagnosis at the onset of *S. maltophilia* infection**					
Arrhythmia	15(25.9)	17(18.0)	13(20.6)	10(22.2)	0.701
Shock	11(19.0)	15(16.0)	11(17.5)	12(26.7)	0.481

Hypoalbuminemia	46(79.3)	75(78.9)	49(77.8)	41(91.1)	0.289
Coma	6(10.3)	19(20.0)	12(19.0)	16(35.6)	0.018
**SOFA scoring**					
Respiratory systems Score ≥ 2	28(48.3)	43(45.3)	40(63.5)	29(64.4)	0.0476
Cardiovascular systems Score ≥ 2	9(15.5)	7(7.4)	15(23.8)	7(15.6)	0.0391
Neurological systems Score ≥ 2	15(25.9)	32(33.7)	22(34.9)	19(42.2)	0.377
Liver system Score ≥ 2	7(12.1)	14(14.7)	11(17.5)	3(6.7)	0.410
Kidney system Score ≥ 2	13(22.4)	16(16.8)	16(25.4)	9(20.0)	0.609
Coagulation system Score ≥ 2	11(19.0)	21(22.1)	12(19.0)	15(33.3)	0.276

Abbreviations: CSL, cefoperazone-sulbactam; FQ, fluoroquinolone; TMP-SMX, trimethoprim-sulfamethoxazole; SOFA, Sequential Organ Failure Assessment.

Notes: *P* values represent overall comparisons across the four treatment groups. No pairwise post-hoc comparisons were performed.

Supplementary Table S2. Summary statistics and percentiles of IPTW weights (two-group analysis)

**Metric**	**Value**
Minimum	1.357
1st quartile (Q1)	1.627
Median	1.740
Mean	2.004
3rd quartile (Q3)	2.298
Maximum	3.798
1st percentile (P1)	1.357
99th percentile (P99)	3.795

Supplementary Figure S1. Distribution of IPTW weights (two-group analysis)

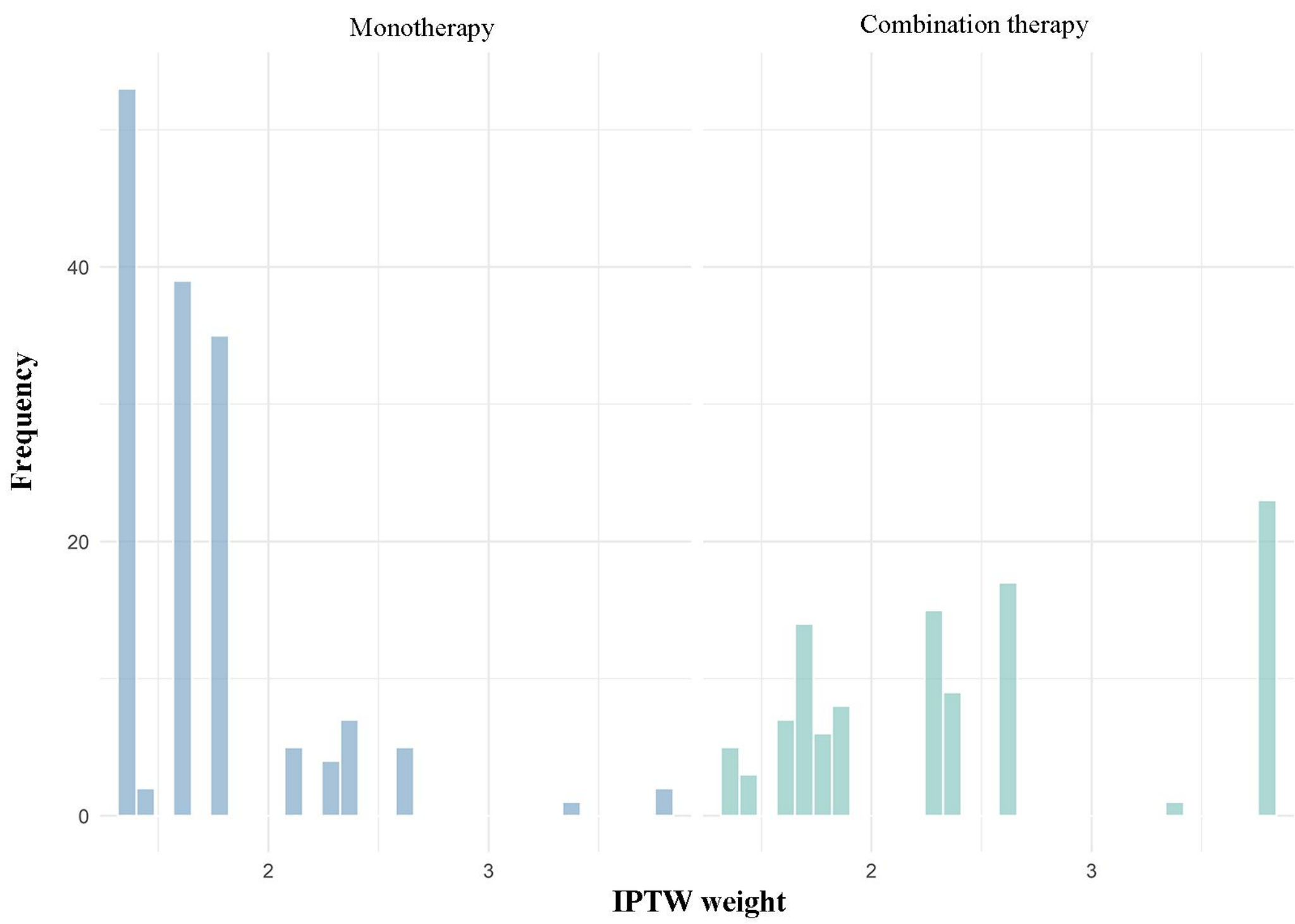

Supplementary Table S3. Distribution of IPTW weights before and after truncation (four-group analysis)

Metric	Before truncation	After truncation (1st–99th percentile)
Minimum	1.736	1.918
1st quartile (Q1)	2.312	2.312
Median	3.306	3.306
Mean	4.072	3.989
3rd quartile (Q3)	4.548	4.548
Maximum	23.480	12.427
1st percentile (P1)	1.918	—
99th percentile (P99)	12.427	—
Truncation bounds	—	1.918–12.427

Notes: IPTW weights were truncated (winsorized) at the 1st and 99th percentiles (lower bound 1.917766; upper bound 12.426737).

Supplementary Figure S2. Distribution of IPTW weights (four-group analysis)

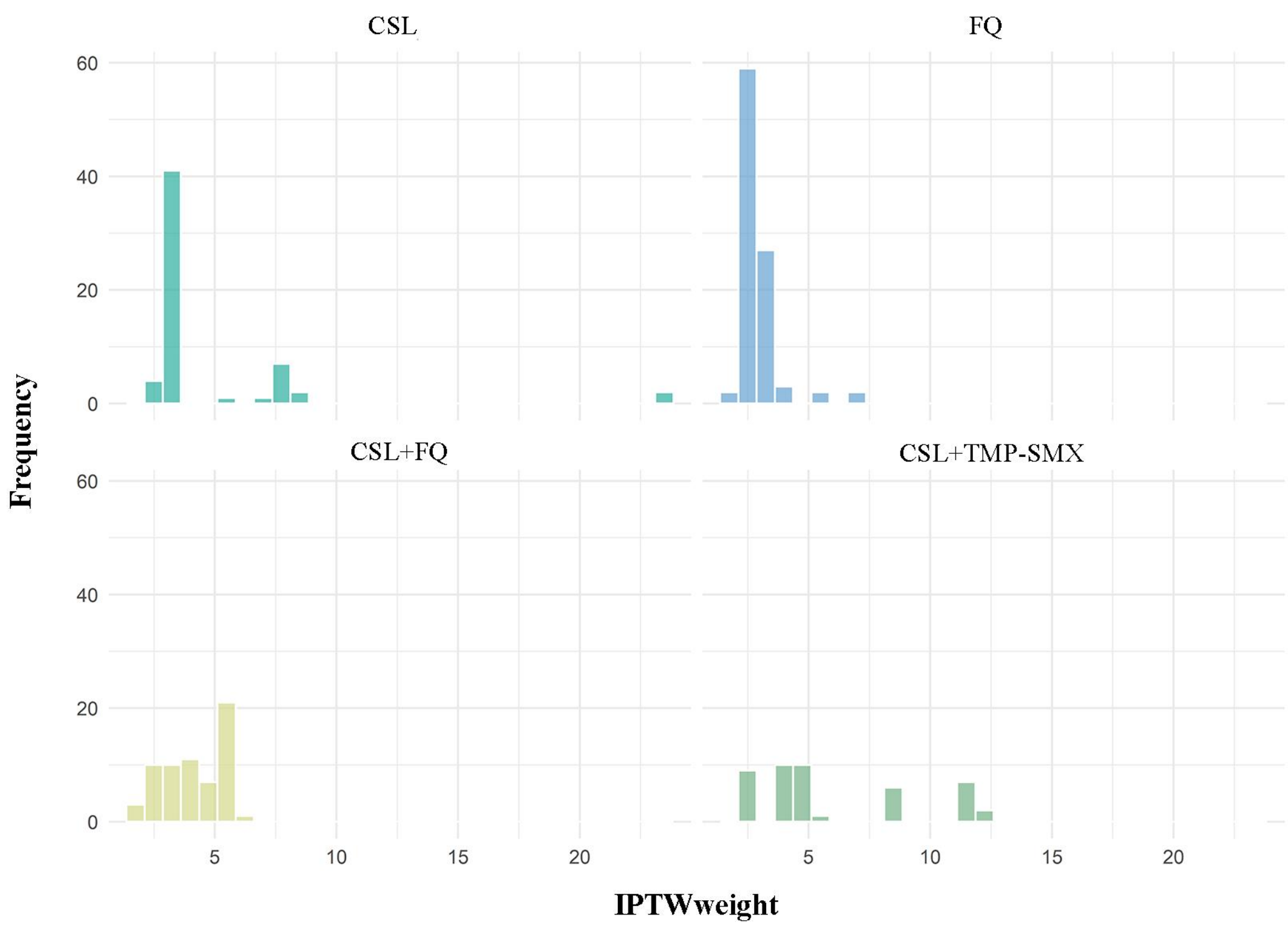

Supplementary Figure S3. Standardized Mean Differences of Baseline Covariates Between Combination Therapy and Monotherapy Groups After IPTW Adjustment

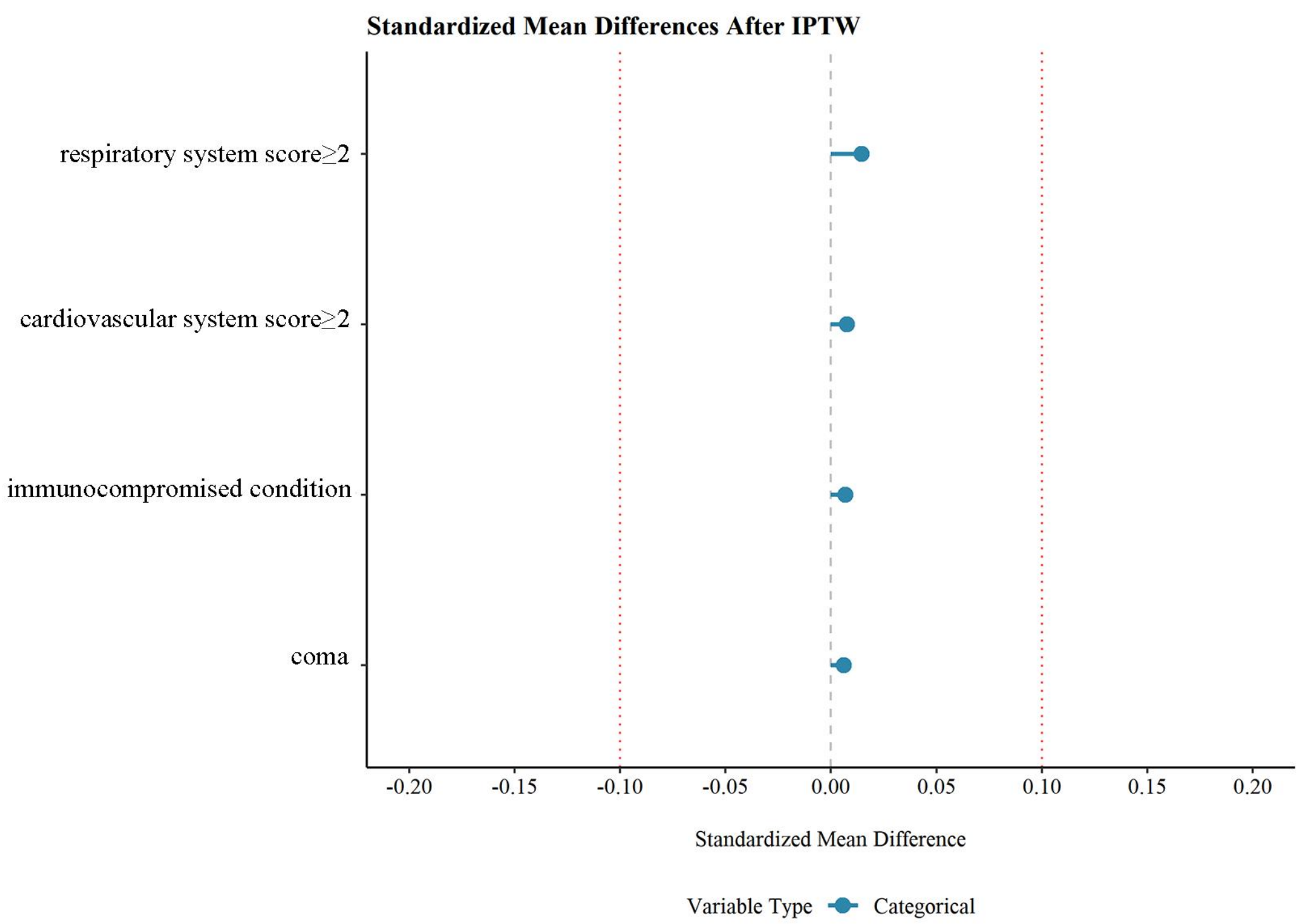

Supplementary Figure S4. Standardized Mean Differences of Baseline Covariates Between Antibiotic Regimens After IPTW Adjustment

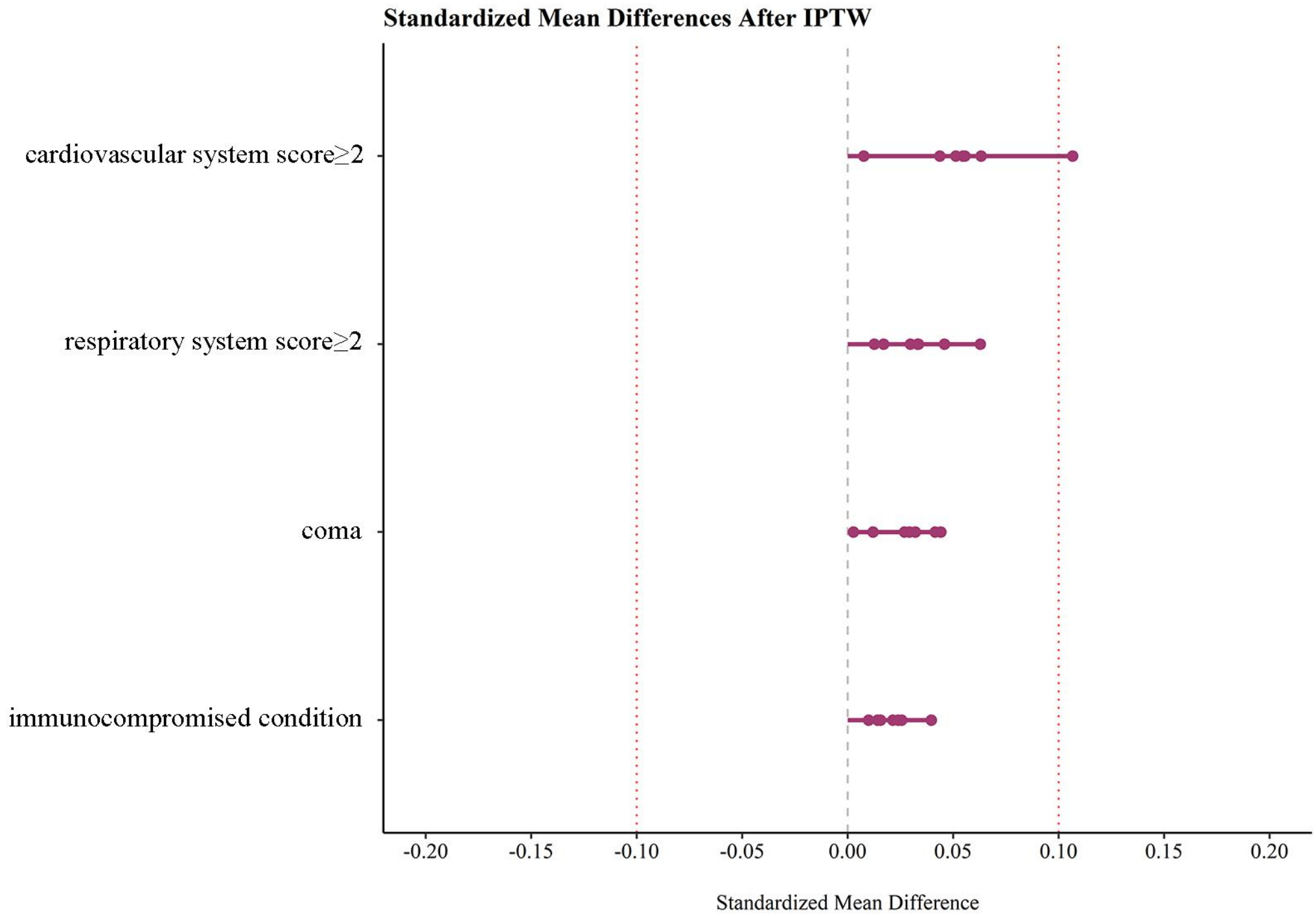

Supplementary Table S4. Sensitivity analysis using stabilized IPTW weights (Combination therapy vs Monotherapy)

**Analysis**	**Weighting approach**	**HR (Combination vs Monotherapy)**	**95% CI**	***P* value**
Primary analysis	Unstabilized IPTW	0.236	0.128–0.434	<0.001
Sensitivity analysis	Stabilized IPTW (SW)	0.235	0.128–0.431	<0.001

Notes: Hazard ratios (HRs) were estimated using IPTW-weighted Cox proportional hazards models, comparing combination therapy with monotherapy. Models were

adjusted for age >60 years, nervous system SOFA score, intubation, respiratory failure, and shock. IPTW indicates inverse probability of treatment weighting; SW indicates stabilized weights; CI indicates confidence interval.

Supplementary Table S5. Sensitivity analysis of weighted Cox models using truncated vs untruncated IPTW weights

**Treatment regimen (vs CSL)**	**Primary analysis (truncated IPTW) HR (95% CI)**	***P* value**	**sensitivity analysis (untruncated IPTW) HR (95% CI)**	***P* value**
FQ	2.345 (1.325-4.149)	0.003	2.313 (1.347-3.972)	0.002
CSL+FQ	0.572 (0.261-1.252)	0.162	0.553 (0.258-1.185)	0.128
CSL+TMP-SMX	0.15 8(0.038-0.653)	0.010	0.153 (0.038-0.619)	0.008

Notes: Primary analysis used IPTW weights truncated (winsorized) at the 1st and 99th percentiles; SA refit the same weighted Cox model using untruncated IPTW weights.

Supplementary Table S6. Antimicrobial Regimens and 28-Day Mortality in the Primary Cohort and Resistance-Restricted Sensitivity Analysis Cohort

**Treatment Approach**	**Regimen**	**Primary cohort, n**	**Primary cohort deaths, n/N (%)**	**Resistance-restricted cohort, n**	**Resistance-restricted cohort deaths, n/N (%)**
Monotherapy	Cefoperazone-sulbactam	58	16/58 (27.6%)	43	12/43 (27.9%)
	Fluoroquinolone (levofloxacin, moxifloxacin, or ciprofloxacin)	95	50/95 (52.6%)	80	44/80 (55.0%)
Combination therapy	Cefoperazone-sulbactam plus fluoroquinolone	63	12/63 (19.0%)	50	9/50 (18.0%)
	Cefoperazone-sulbactam plus trimethoprim-sulfamethoxazole	45	3/45 (6.7%)	35	2/35 (5.7%)

Total	—	261	81/261 (31.0%)	208	67/208 (32.2%)

Note: The resistance-restricted cohort included patients whose isolates were susceptible in vitro to cefoperazone-sulbactam, fluoroquino lones, and trimethoprim-sulfamethoxazole and who had no documented contraindications to these agents.

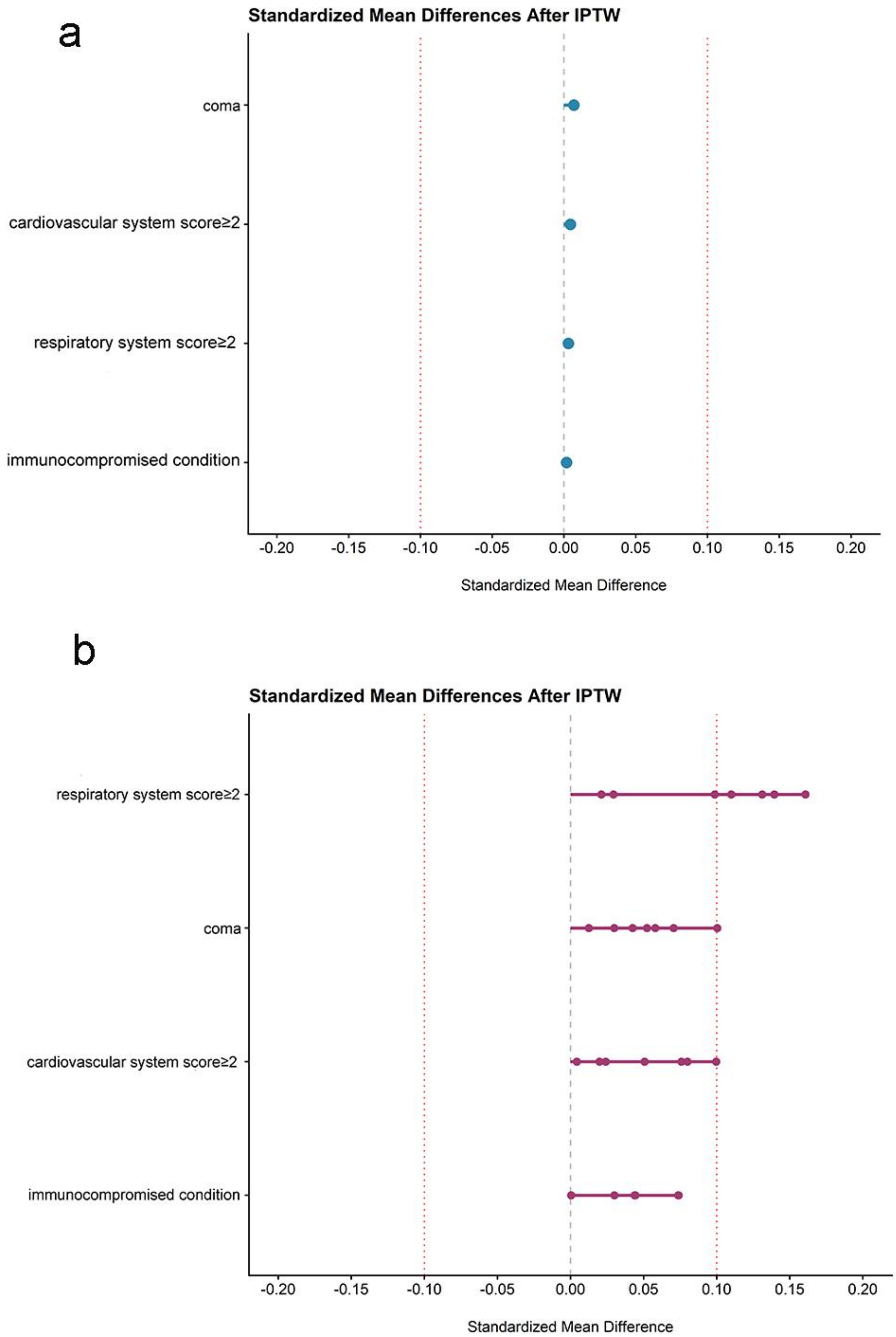

Supplementary Figure S5. Standardized mean differences after IPTW adjustment in the resistance-restricted sensitivity analysis cohort.

(a) Standardized mean differences of baseline covariates between the monotherapy and combination therapy groups after IPTW adjustment.

(b) Standardized mean differences of baseline covariates across the four antibiotic regimens after IPTW adjustment. Most post-weighting SMDs were <0.1, although respiratory system SOFA score ≥2 remained slightly above the prespecified threshold.

Supplementary Table S7. Sensitivity Analysis Comparing IPTW-Weighted Cox Regression Results in the Primary and Resistance-Restricted Cohorts

**Analysis**	**Primary cohort HR (95% CI), *P* value**	**Resistance-restricted cohort HR (95% CI), *P* value**
Combination vs monotherapy	0.236 (0.128–0.434), *P*<0.001	0.236 (0.118–0.472), *P*<0.001
FQ vs CSL	2.345 (1.325–4.149), *P*=0.003	2.344 (1.215–4.521), *P*=0.010
CSL+FQ vs CSL	0.572 (0.261–1.252), *P*=0.162	0.509 (0.206–1.258), *P*=0.143
CSL+TMP-SMX vs CSL	0.158 (0.038–0.653), *P*=0.010	0.204 (0.041–1.003), *P*=0.050

Supplementary Table S8. Time-related variables across the four antimicrobial regimen groups

**Time-related variable**	**CSL monotherapy**	**FQ monotherapy**	**CSL+ FQ**	**CSL+ TMP-SMX**	***P* value**
Time from hospital admission to first positive culture collection, days	12 (3–17)	9 (3–17.5)	13 (5–18.5)	13 (4–22)	0.486
Time from culture collection to antimicrobial suscepti bility reporting, days	3 (2–3)	2 (2–3)	3 (2–3)	3 (2–3)	0.050
Days of use of any definitive-regimen agent before s usceptibility reporting	2.5 (1–3)	3 (2–3)	3 (1–4)	2 (1–3)	0.167
Any definitive-regimen agent initiated before susceptib ility reporting, n/N (%)	9/58 (15.5%)	8/95 (8.4%)	6/63 (9.5%)	10/45 (22.2%)	0.100

Abbreviations: CSL, cefoperazone-sulbactam; FQ, fluoroquinolone; TMP-SMX, trimethoprim-sulfamethoxazole.

Note: Continuous variables are presented as median (interquartile range). Categorical variables are presented as n/N (%). *P* values for

continuous variables were calculated using the Kruskal–Wallis test, and *P* values for categorical variables were calculated using the Pearson chi-square test.
